# Restoring disc matrix homeostasis: Dual-miRNA and human platelet lysate as a novel therapeutic strategy

**DOI:** 10.1016/j.mtbio.2026.103190

**Published:** 2026-05-03

**Authors:** Tara Ní Néill, Niamh Wilson, Jijo Thomas, Jake McDonnell, Stacey L. Darwish, Joseph S. Butler, Fergal J. O'Brien, James E. Dixon, Caroline M. Curtin, Conor T. Buckley

**Affiliations:** a Trinity Centre for Biomedical Engineering, Trinity Biomedical Sciences Institute, Trinity College Dublin, The University of Dublin, Ireland; b Discipline of Mechanical, Manufacturing and Biomedical Engineering, School of Engineering, Trinity College Dublin, The University of Dublin, Ireland; c Advanced Materials and Bioengineering Research (AMBER) Centre, Royal College of Surgeons in Ireland & Trinity College Dublin, The University of Dublin, Dublin, Ireland; d National Spinal Injuries Unit, Mater Misericordiae University Hospital, Dublin, Ireland; e School of Medicine, University College Dublin, Ireland; f Department of Orthopaedics, St Vincent's University Hospital, Dublin, Ireland; g Tissue Engineering Research Group, Department of Anatomy and Regenerative Medicine, Royal College of Surgeons in Ireland, Dublin, Ireland; h Regenerative Medicine and Cellular Therapies, The University of Nottingham Biodiscovery Institute (BDI), School of Pharmacy, University of Nottingham, Nottingham, UK; i NIHR Nottingham Biomedical Research Centre, University of Nottingham, Nottingham, UK

**Keywords:** microRNA, Human platelet lysate, Intervertebral disc degeneration, Ex vivo organ culture

## Abstract

Intervertebral disc (IVD) degeneration is the predominant cause of low back pain, resulting from progressive extracellular matrix (ECM) degradation and establishment of a pro-catabolic microenvironment. microRNA (miRNA) delivery has potential to promote ECM restoration and curb the catabolic milieu. We previously demonstrated a robust anti-catabolic effect through the delivery of a dual-miRNA injectable therapy in ex vivo organ culture models of degeneration. However, to further enhance regenerative outcomes, additional stimulation, such as supplementation with human platelet lysate (HPL) may strengthen this therapeutic strategy. Supplementation of rat nucleus pulposus (NP) cells with the dual-miRNA-FLR-149-5p mimic and miRNA-221-3p inhibitor and HPL was assessed in monolayer and ex vivo organ culture modelling mild degeneration. To evaluate the translatability of our approach, a tuneable patient NP cell-laden NP-ECM gel analogue was developed. For all models, key ECM constituents and matrix-degrading proteins were assessed biochemically, histologically, and using immunofluorescence. HPL supplementation combined with FLR-miRNA-149-5p and 221-3p inhibitor resulted in increased ECM regenerative proteins, SRY-box transcription factor 9 (SOX9), and aggrecan, while suppressing catabolic factors from matrix-degrading enzymes disintegrin and metalloproteinase with thrombospondin motifs (ADAMTS) and matrix metalloproteinases (MMPs). Importantly, rat and human culture models were consistent in their response, highlighting the clinical translatability of our models and therapeutic strategy. Combined delivery of dual-miRNA with HPL delivery in rat and human models of mild degeneration enhanced expression of restorative ECM proteins while suppressing matrix-degrading factors. These findings highlight a novel and promising therapeutic strategy for the treatment of early-stage IVD degeneration.

## Graphical abstract

**Figure:**
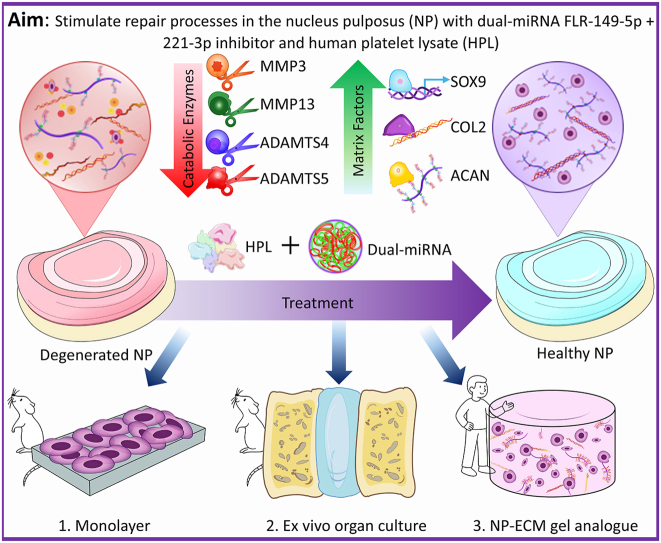

## 1 Introduction

Degeneration of the intervertebral disc (IVD) is the leading cause of low back pain worldwide, affecting 619 million people in 2020, with this number projected to increase to 843 million by 2050 [1]. The IVD is an avascular tissue comprised of three main regions: the central nucleus pulposus (NP), enclosed by the lamellae of the annulus fibrosus (AF) and bounded proximally and distally by the cartilaginous endplate (CEP) [2]. This work focuses on the NP, due to its crucial role in maintaining IVD integrity and function, with its degeneration being a key driver of disc deterioration and the onset of low back pain [3]. Though pleiotropic in nature, matrix dysregulation, imbalances between anabolic and catabolic factors, and tissue disruption are key components of the degenerative cascade [4,5]. This results in a hostile microenvironment characterised by impaired solute transport and a limited regenerative capacity, creating a challenging setting for regenerative therapeutics [[6], [7], [8]]. This is reflected by the paucity of options currently available beyond pain management, conservative treatments, and surgical intervention, highlighting a critical unmet clinical need [9].

A multitude of regenerative medicine approaches have been trialled, including cell therapies [10,11], biomaterial augmentation [12,13], and growth factor (GF) delivery [[14], [15], [16]]. In pre-clinical models, GF supplementation appears to be promising and beneficial, but the long-term efficacy has been limited, particularly due to single-dose durability and short half-life [17,18]. Building on this, instead of incorporating a single factor, using a cocktail may offer a multi-pronged strategy to enhance regenerative outcomes. Platelet lysate, derived from plasma, is enriched with multiple GFs including but not limited to members of the transforming growth factor (TGF) family, platelet-derived growth factor (PDGF), and vascular endothelial growth factor (VEGF) [19]. Originally used as a serum substitute in cell culture [20], platelet lysate has demonstrated extracellular matrix (ECM) enhancing potential in the field of orthopaedics, including the upregulation of key components such as aggrecan and collagen type II, both in vitro and in vivo [[21], [22], [23]].

Human platelet lysate (HPL) delivery has been explored clinically in the treatment of numerous musculoskeletal conditions, involving tissues with limited intrinsic healing potential, similar to the IVD. In the IVD field, a preliminary prospective trial reported that 47% of patients had significant improvements to the visual analogue score (VAS) and Oswestry Disability Index (ODI) 6 months-post single platelet rich plasma injection [24]. A prospective double-blind randomised clinical trial (RCT) evaluated a single intradiscal lysate injection in patients with chronic low back pain, reporting statistically significant improvements in pain, function, and patient satisfaction compared to the control group 8 weeks post-treatment [25]. Akeda and colleagues also reported significant improvements to patient VAS scores following a single intradiscal injection of platelet-rich plasma, though, interestingly no improvements in disc height index were observed [26]. However, it should be noted that evidence from the disc field is sparse, detailing changes to pain scores but often no structural or mechanical changes. Importantly, none of these studies have reported adverse effects associated with lysate administration, highlighting its safety and translational potential as a low risk, first-line therapeutic option in orthopaedics.

Effective IVD therapies will likely augment and promote ECM production while mitigating the inflammatory and catabolic responses. Incorporating an anti-catabolic factor offers a multimodal strategy whereby inflammation is suppressed, allowing matrix regeneration to proceed. MicroRNAs (miRNAs) are small, non-coding nucleic acids, capable of modulating the expression of thousands of genes and their corresponding proteins, making them an attractive therapeutic avenue [[27], [28], [29], [30]]. Our laboratory has previously investigated the co-delivery of an anti-catabolic dual-miRNA, miRNA-149-5p mimic and miRNA-221-3p inhibitor using the cell penetrating glycosaminoglycan (GAG)-binding enhanced transfection (GET) FLR peptide as a delivery vector [31,32]. We have demonstrated significant downregulated expression of the matrix degrading enzymes disintegrin and metalloproteinase with thrombospondin motifs 5 (ADAMTS5) and matrix metalloproteinase 13 (MMP13), using both in vitro and ex vivo models. The FLR peptide is of particular relevance to the IVD, due to a fibroblastic growth factor 2 (FGF2) heparin binding domain that specifically homes to GAG-rich areas such as NP tissue which is rich in proteoglycans [[33], [34], [35]]. Given the promising anti-catabolic action of miRNA delivery and the regenerative potential of platelet lysate, we proposed supplementing our dual-miRNA to enhance matrix production. The primary objective of this work was to investigate the potential of dual-miRNA delivery supplemented with HPL in a range of culture models and species, to identify whether the anti-catabolic effect was maintained whilst driving the production of ECM proteins. We hypothesised that the combined delivery of our dual-miRNA and HPL may stimulate the repair process, offering a novel prospective treatment for disc degeneration.

## 2 Materials and methods

### 2.1 Primary rat and human nucleus pulposus cell isolation

Rat caudal tissue was obtained from discarded tissue of animals undergoing procedures in the Comparative Medicines Unit (CMU) of Trinity College Dublin, with approval from the animal research ethics committee (AREC) of Trinity College Dublin and the Health Products Regulatory Authority in Ireland (HPRA, Approval – AE19136/P149). Briefly, immediately following euthanasia, three tails from mature female Wistar rats were pooled, and the NP tissue excised under sterile conditions. Enzymatic digestion was performed simultaneously with pronase 70 U/mL, Millipore) and collagenase type II (400 U/mL, Gibco) for 6 h at 37 °C at 10 rpm [36].

Human NP cells were isolated from disc tissue collected with informed consent from patients undergoing discectomy procedures, approved by the Mater Misericordiae University Hospital, Dublin Institutional Review Board (Ref [1]/378/2229) and Trinity College Dublin (TCDFSTEMSREC/15032021). For in vitro experiments cells from 3 females and 2 males with mild to moderate degeneration as assessed by Pfirrmann grading [37] were utilised, ranging in age from 24 to 57, outlined in Table 1. To ensure sterility, tissue was incubated overnight in serum free (SF) Dulbecco's Modified Eagle Medium - low glucose (DMEM LG, Sigma-Aldrich) with 2% penicillin/streptomycin (P/S, Gibco), 100 μg/mL kanamycin sulphate (Gibco), and 2.5 mg/mL Amphotericin B (Sigma-Aldrich). NP tissue was dissected from the remaining disc tissue under sterile conditions and digested enzymatically with 100 U/mL pronase and 300 U/mL collagenase type II. Following trituration to obtain a single cell suspension, cells were plated at a density of 5 × 10^3^ cells/cm^2^ and expanded to confluency in expansion media (XPAN), containing DMEM LG supplemented with 2% P/S and 10% foetal bovine serum (FBS, all Gibco). Cells were maintained at physioxic (5% O_2_) conditions in a humidified atmosphere at 37 °C, with media measuring between 300 and 325 mOsm, correlating to the osmolarity of a degenerated disc [38].

**Table 1:** Demographic and clinical characteristics of human donors used for in vitro studies.

Donor	Gender	Age	Smoker (Y/N)	Pfirrmann Grade
1	F	24	No	3
2	M	44	Former – 15 years	2
3	F	29	No	2
4	M	57	No	3
5	F	33	No	3

### 2.2 HPL utilised throughout all experiments

To ensure consistency between experiments, commercially available allogeneic HPL from the same batch number was employed. HPL was collected from human platelets at U.S Food and Drug Administration (FDA)-licensed blood centres. The HPL was pooled from multiple donors under good manufacturing practice (GMP) conditions (StemCell Technologies, Canada). The total protein content of the HPL was between 4.0 and 8.0 g/dL. To evaluate the levels of key GFs, ELISAs were performed for TGF-β, VEGF, and PDGF (HUFI00248, HUFI00281, and HUFI02970, respectively, all Assay Genie) according to manufacturer's instructions.

### 2.3 HPL dosage study in monolayer

Rat NP cells were seeded in microwell chamber slides (ibidi GmbH) at a density of 5 × 10^3^ cells per well in XPAN, where attachment was allowed to proceed overnight and subsequently washed with phosphate buffered saline (PBS). Media was added, either SF DMEM LG alone or supplemented with 2.5, 5, or 10% of HPL. Cells were cultured for 3 days and fixed in 4% paraformaldehyde (PFA) for 12 min followed by assessment of aggrecan and collagen type II protein expression. The extent of cell proliferation was assessed by counting the nuclei from each group. For all studies a minimum of three independent biological replicates derived from distinct donors were performed, with a minimum of duplicate technical replicates. Biological replicates are indicated by individual data points in the graphs.

### 2.4 Confirmation of miRNA over- and under-expression via RT-qPCR

To confirm the successful delivery of miRNA-149-5p mimic and miRNA-221-3p inhibitor, rat NP cells were seeded at a density of 10 × 10^4^ in 48-well plates and allowed to attach overnight. miRNA complexes of 10 ng/μl miRNA-149-5p mimic and miRNA-221-3p inhibitor (both Dharmacon) with the FLR peptide (Dixon Lab, Nottingham) were formed via electrostatic interactions in OptiMEM reduced serum media (Gibco) for 30 min. Cells were washed with PBS to remove residual serum and transfected for 6 h at 37 °C at 5% O_2_. Post-transfection wells were replenished with XPAN media and cultured for 3 days for analysis of miRNA over- and under-expression. Total RNA was extracted using QIAzol reagent and the miRNeasy kit (Qiagen) according to manufacturer's instructions. The miRCURY LNA reverse transcription kit and validated miRCURY LNA SYBR Green PCR assays were utilised to assess levels of miRNA-149-5p and miRNA-221-3p (all Qiagen). Relative expression levels were normalised to the endogenous 18S ribosomal RNA and calculated using the 2(-ΔΔCt) method to compare expression levels between non-transfected (NT) and transfected cells. Previous work in our laboratory had independently confirmed that scrambled miRNA and FLR only delivery had no functional effect upon cells in vitro or ex vivo, therefore these groups were not included in this study [31,32].

### 2.5 Dual-miRNA delivery supplemented with HPL in monolayer

Upon fixing the HPL dosage, the effect of dual miRNA delivery augmented with HPL was examined. NP cells were seeded at a density of 5 × 10^3^cells in microwell chamber slides (ibidi GmbH) and allowed to attach overnight. Transfection was performed as described above, where following transfection SF DMEM LG media, or DMEM LG + 10% HPL media was applied to cells, which were cultured for 3 days for protein analysis of ECM markers, aggrecan and collagen type II, alongside proliferation analysis. Stimulation of matrix degrading enzymes was achieved through a cytokine challenge 24 h post-transfection of 50 ng/mL TNF and 10 ng/mL IL-1β (both Fisher Scientific). Cells were insulted for 24 h whereupon media was replaced as described above and culture proceeded for 3 days.

### 2.6 Induction of degeneration in a rat ex vivo organ culture model

Tails from female Wistar rats were procured from discarded tissue and prepared as previously described [31,36]. In short, excess fascia and tendons were removed from one side of the tail, leaving the caudal disc exposed. To maintain consistency in disc size, the top three motion segments were isolated and cultured overnight in antibiotic supplemented media (2% P/S, Amphotericin B (0.1 μl/mL, and Kanamycin (100 μg/mL, Gibco)). Mild degeneration of the NP was induced by injecting 2 μl of 0.025 U chondroitinase ABC (cABC, Sigma-Aldrich) through a 30G needle, followed by culture for 7 days to allow degeneration to progress. For all experiments a non-degenerated control (no cABC treatment) was included.

### 2.7 Assessment of miRNA and HPL treatment in rat ex vivo organ culture

To confirm the viscosity of HPL did not adversely affect uptake of the dual-miRNA, a fluorescently-tagged miRNA miRDy547 (Dharmacon) was first employed. Following degeneration, miRDy547 complexes (25 ng/μl) were prepared by incubating with FLR in OptiMEM for 30 min at room temperature. Where applicable, 10% HPL was subsequently incorporated and 2 μl of the mixture was delivered to rat discs. Uptake of the fluorescently tagged miRNA was assessed after 7 days of culture. Treatments were prepared and administered in 2 μl volumes using a 30G needle. The non-transfected (NT) control received OptiMEM as the vehicle, while the HPL-only group received 10% HPL prepared in the same vehicle. Dual-FLR-miRNA-149-5p mimic and miRNA-221-3p inhibitor complexes were prepared at a concentration of 25 ng/μl in OptiMEM as optimised from previous studies [31,32] and incubated for 30 min at room temperature. miRNA complexes were directly injected for the miRNA-only group, whereas for the miRNA + HPL group, 10% HPL was added to the complexes prior to injection. Treatments were maintained in culture for 14 days with biweekly media exchanges performed. Collected media was pooled to assess GAG released during the culture period. At the end of culture period, discs were fixed overnight with 4% PFA at 4 °C for histological evaluation, or the NP region was excised for cell viability analysis, biochemical assays and cytokine quantification.

### 2.8 NP-ECM, methacrylated NP-ECM (NP-ECM-MA), and methacrylated chondroitin sulphate (CS-MA) preparation

To simulate ex vivo organ culture while integrating human patient-derived cells, an NP biomimetic ECM analogue was engineered. Fresh bovine NP tissue was dissected and finely diced into approximately 2 mm fragments. The tissue was then pretreated with 0.2 M NaOH for 24h at 4 °C under constant rotation to assist with subsequent tissue digestion. The tissue was washed with PBS sequentially prior to freeze drying and cryo-milled with a cryogenic grinder (6775 Freezer/Mill, SPEX SamplePrep) to obtain a fine NP-extracellular matrix (ECM) powder. Nucleic acids were removed through treatment with Benzonase nuclease (2U/mL, Sigma-Aldrich) in Tris Buffer. Solubilisation was carried out with activated 18000 U/mL pepsin solution in 0.5 M acetic acid (HAc) for 48 h at room temperature under constant rotation. Salt precipitation was performed using 0.9 M NaCl followed by dialysis against deionised water with 12-14 kDa MWCO dialysis membrane at 4 °C. Dialysis was carried out for 48 h with water changes performed twice daily. The dialysate was freeze dried under standard conditions (500 mTorr, −10 °C, 16 – 18h, Harvest Right™) and stored frozen (−20 °C) until further use.

Methacrylate groups were added to the NP-ECM to facilitate photo-crosslinking. To begin, NP-ECM was solubilised in 2.5 mg/mL 0.5M HAc overnight at 4 °C with vigorous agitation, whereupon the pH was adjusted to 7.5. Protected from light, methacrylic anhydride was added at a dose of 20 μl per 100 mg of NP-ECM in a dropwise fashion and stirred overnight at 4 °C. NP-ECM-MA was dialysed in 12-14 kDa MWCO membranes, initially against 1% v/v HAc for 3 days at 4 °C, followed by deionised water for a further 3 days. For each dialysis step, the required solution was changed twice per day. The retained NP-ECM-MA was freeze-dried and stored at −20 °C prior to gel preparation. For the functionalisation of chondroitin sulphate (CS), sodium salt (Biosynth) was dissolved in deionised water (20 mg/mL), with 1 mg/mL of methacrylic anhydride added dropwise under constant stirring and protected from light. The solution was adjusted to pH 8 and stirred at room temperature for 3 h, followed by overnight stirring at 4 °C. The unreacted methacrylic anhydride was precipitated out of solution by the addition of a 4-fold excess of ethanol, and the ethanol washing step was repeated three times. CS-MA was resuspended in deionised water, dialysed for 3 days against deionised water, followed by freeze-drying to obtain a CS-MA powder.

### 2.9 Preparation of human NP cell-laden NP-ECM-MA CS-MA analogues

To prepare the ECM analogues, NP-ECM-MA (15% w/v) and CS-MA (10% w/v) powders were weighed and solubilised in XPAN media for 48 h at 4 °C. Prior to cell incorporation, the solutions were mixed at 1:1 ratio to yield a final concentration of 7.5% w/v NP-ECM-MA and 5% w/v CS-MA, corresponding to the matrix composition of a mildly degenerated, Pfirrman Grade 3 disc [39]. The solutions were neutralised with 5M NaOH, and sterilised by ultraviolet (UV) radiation for 15 min. Human NP cells from patient samples ranging from mild to moderately degenerated discs were expanded (passage 3) and utilised. Cells were encapsulated into the gels through mixing at a concentration of 2.5 × 10^6^ cells/mL, which relates to approximately the total number of NP cells found in a degenerated disc [40]. 0.25% v/v lithium phenyl-2, 4, 6-trimethylbenzoylphosphinate (LAP, Sigma-Aldrich) was employed as the photo-initiator and the mixture was injected into 50 μL moulds and crosslinked for 60s under blue light (405 nm). The formed gels were cultured in 4% agarose wells prepared in 48 well plates, each containing an internal well that housed individual gels to facilitate easier injection delivery for subsequent experiments. Culture conditions were maintained in XPAN media at 5% O_2_ in a humidified incubator at 37 °C.

### 2.10 Evaluation of miRNA and HPL treatment using a human cell-laden NP-ECM gel analogue

**Fig. 1:**
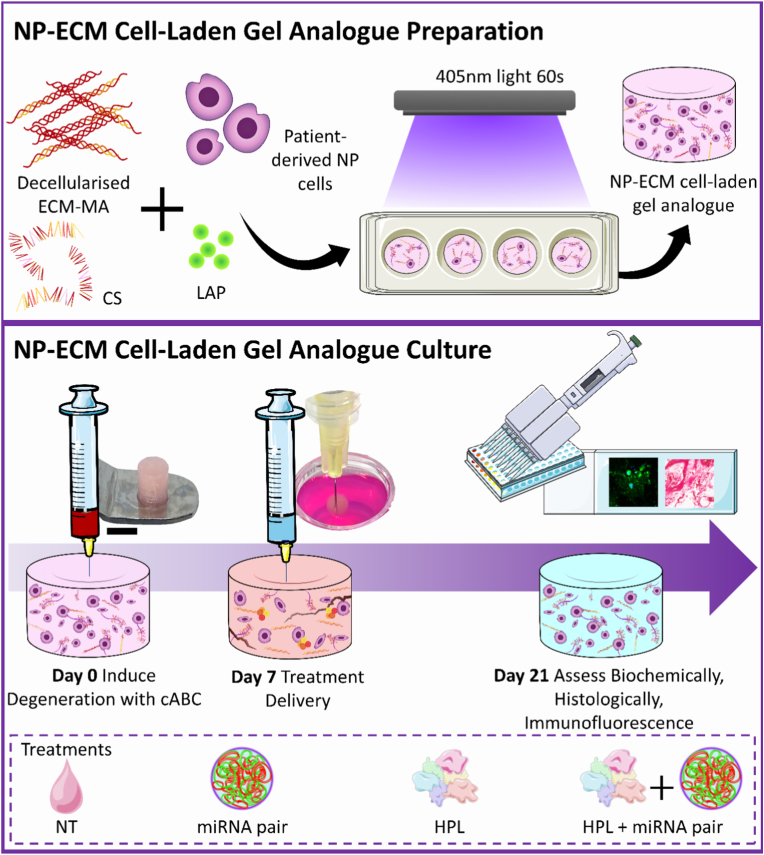
**Experimental outline for generating human cell-laden nucleus pulposus (NP)-extracellular matrix (ECM) gel analogues.** Gel analogues were prepared as described in Section 2.8, and degeneration was induced in samples designated for treatment with chondroitinase ABC (cABC) which cleaves sulphated glycosaminoglycans (sGAG) and triggers a catabolic response. Healthy, non-degenerated controls were included in all experiments. On day 7, gel analogues were injected with the respective treatments and cultured until day 21, when they were analysed biochemically, histologically, and via immunofluorescence for specific anabolic and catabolic markers. Abbreviations: MA, methacrylic anhydride; CS, Chondroitin sulphate; LAP, lithium phenyl-2, 4, 6-trimethylbenzoylphosphinate; NT, Non-transfected. Scale bar = 5 mm.

An overview of the experimental outline for NP cell-laden ECM analogues is presented in Fig. 1. To induce a mildly catabolic and GAG-degrading response comparable to that observed in our rat ex vivo organ culture model, human NP cell-laden gel analogues were injected with 2 μl of 0.025U cABC and cultured for 7 days to allow degeneration to proceed. Following this, degenerated groups were treated with either the vehicle (NT control), FLR-miRNA-149-5p mimic + miRNA-221-3p inhibitor (dual-miRNA), 10% HPL, or 10% HPL + dual-miRNA and cultured for a further 14 days. As an additional healthy control, one group of gels was not treated with cABC for the 21-day culture period. Media changes were performed twice weekly, with the collected media pooled at each time point for biochemical analysis of leached ECM components and ELISA assessment of inflammatory markers. Whole gels were also reserved for biochemical analysis, while those designated for histology were fixed in 4% PFA overnight at 4 °C.

### 2.11 Assessment of cell viability in rat ex vivo organs and NP analogues

At the culture endpoints, the entire NP tissue was dissected from rat ex vivo organ culture motion segments. The viability of both NP tissue and human cell-laden NP-ECM gel analogues was assessed through staining with 2 μM Calcein AM (Sigma-Aldrich) and 4 μM ethidium homodimer (EthD-1, SLS Laboratory Supplies) for 1 h at 37 °C. Samples were imaged on a Leica SP8 scanning confocal microscope, where a z-stack of the entire NP tissue or NP analogue was taken (485 nm excitation, 530 nm emission for Calcein, 530 nm excitation, 645 nm emission for EthD-1). Acquired images were analysed using ImageJ software (version 1.54p) to identify the live and dead cell populations.

### 2.12 Biochemical analysis of ex vivo organ culture and human cell-laden NP-ECM gel analogues

At the experimental endpoints, the NP region was excised from each disc and subjected to papain digestion. Likewise for the human cell-laden NP-ECM gel analogues, intact gels were carried forward for papain digestion. Briefly, the tissues and gels were incubated for 18 h at 60 °C in 100 mM sodium phosphate/5 mM Na_2_EDTA buffer with 3.88 U/mL papain enzyme (all Merck Life Science Ltd.). DNA quantification was performed with the Hoechst Bisbenzimide 33258 dye assay (Merck Life Science Ltd.) with a calf thymus DNA standard. Sulphated glycosaminoglycan levels were determined using a dimethylmethylene blue (DMMB) assay with a chondroitin sulphate standard. Finally, total collagen content was assessed through a hydroxyproline assay, whereby samples were hydrolysed in 38% concentrated hydrochloric acid at 110 °C for 18 h, followed by a chloramine-T assay. A hydroxyproline-to-collagen ratio of 1:7.69 was used to calculate collagen levels [41].

### 2.13 Histology and immunocytochemistry

After overnight fixation with 4% PFA, rat discs were decalcified with Decalcifying Solution-Lite (Merck Life Science Ltd.) for approximately 7 days until softened, then sequentially dehydrated through increasing ethanol concentrations and embedded in paraffin. Sections of 10 μm were cut using a microtome (Leica). To preserve structural integrity, human cell-laden NP-ECM gel analogues were immersed in 30% sucrose as a cryoprotectant, embedded in optimal cutting temperature (OCT) compound, and snap-frozen in liquid nitrogen. Cryo-sections of 7 μm were obtained using a Leica CM3050 S cryostat. Histological staining was performed as follows: haematoxylin and eosin (H&E) for cellularity, picrosirius red (PR) for collagen, and alcian blue (AB) for sGAG deposition. Sections were imaged on an Aperio AT2 Slide Scanner (Leica).

For immunofluorescence of paraffin wax embedded samples, antigen retrieval was performed in sodium citrate buffer at pH 6 in a 100 °C pressure cooker for 1 min. For monolayer, ex vivo organ cultures and NP analogue sections, membranes were permeabilised with 0.1% Triton-X for 20 min and blocked in 5% bovine serum albumin in PBS-Tween for 1 h. Primary antibodies (Table 2) were applied overnight at 4 °C. After washing, secondary antibodies were incubated for 2 h at room temperature with nuclear counterstaining using Hoechst 33342. Monolayer and sectioned slides were imaged on a Leica Scanning SP8 confocal microscope, and mean fluorescence intensity measurements of all experiments were normalised to cell number. For all fluorescence experiments a background correction was applied to ensure accurate quantification of rat ex vivo NP tissue and human NP cell-laden NP-ECM analogues.

**Table 2:** Primary and secondary antibodies used for immunohistochemical staining with heat induced epitope retrieval (HIER).

Antigen	Species	Concentration	Supplier & Code	Antigen Retrieval Method
IgG	Rabbit	1:1250	Invitrogen, 26102	HIER
IgG	Mouse	1:1250	Invitrogen, 31903	HIER
Aggrecan	Rabbit	1:250	Invitrogen, MA532695	HIER
SOX9	Rabbit	1:250	Invitrogen, PA586301	HIER
Collagen type I	Rabbit	1:100	Invitrogen, PA529569	HIER
Collagen type II	Rabbit	1:250	Invitrogen, PA599159	HIER
ADAMTS4	Rabbit	1:75	Invitrogen, PA585211	HIER
ADAMTS5	Rabbit	1:250	Invitrogen, PA532142	HIER
MMP3	Rabbit	1:125	Invitrogen, 710309	HIER
MMP13	Mouse	1:125	Invitrogen, MA514247	HIER
Anti-Rabbit Alexa Fluor 488	Goat	1:500	Invitrogen, A27034	N/A
Anti-Mouse Alexa Fluor 488	Goat	1:2000	Invitrogen, A11001	N/A

### 2.14 Statistical analysis

Statistical analyses were performed using GraphPad Prism version 10.4.0. Outlier and normality tests were performed for each dataset. Comparisons between two groups were performed using unpaired t-tests, while comparisons among three or more groups were analysed using one-way ANOVA with Tukey's post-hoc test. A p-value of ≤ 0.05 was considered statistically significant, and is denoted by indicators ∗ p < 0.05, ∗∗p < 0.01, ∗∗∗p < 0.001, ∗∗∗∗p < 0.0001. p-values ≤ 0.1 are indicated on all relevant graphs to denote potential trends, while p-values > 0.1 for all group comparisons are provided in Supplementary Table 1. All experiments included a minimum of three independent biological donors, represented as individual datapoints in the graphs. To account for donor variance, particularly from human cells, all data is normalised to the relevant negative control. Graphical data is presented as mean ± standard deviation (SD).

## 3 Results

### 3.1 Confirmation of HPL dosage and miRNA expression in rat nucleus pulposus monolayer culture

**Fig. 2:**
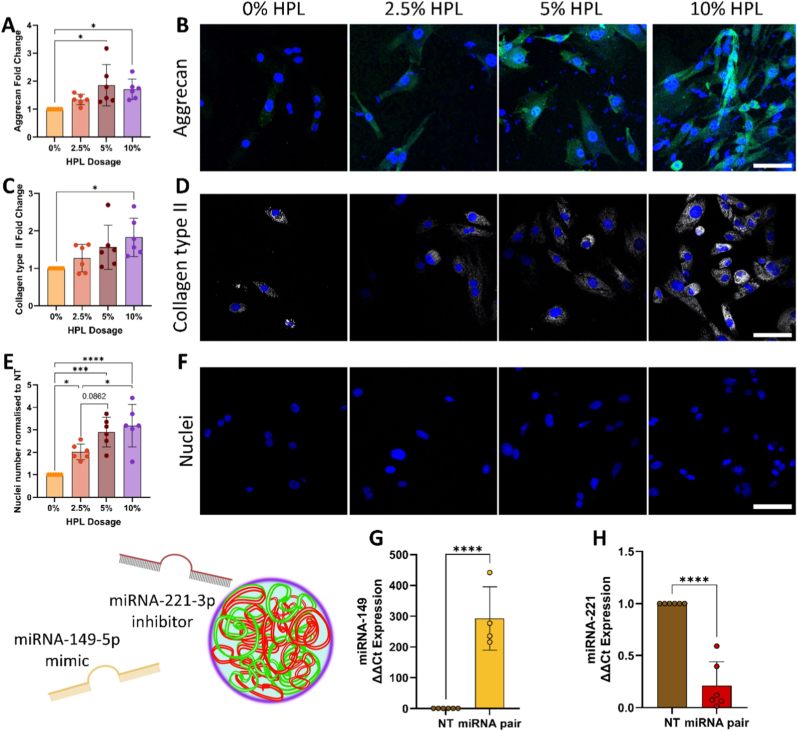
**Increasing doses of human platelet lysate (HPL) increased extracellular matrix markers, alongside cell proliferation in rat nucleus pulposus (NP) monolayer culture.** Protein expression was quantified via immunofluorescence, with representative images displayed for (A & B) aggrecan (green) and (C & D) collagen type II (grey). (E & F) The number of nuclei were assessed through DAPI staining. (G & H) RT-qPCR was used to assess the expression of miRNA-149 and miRNA-221 3 days post-transfection. N = 6 independent biological donors, immunofluorescence data normalised to 0% HPL control, RT-qPCR to the non-transfected (NT) control. ∗p < 0.05, ∗∗∗p < 0.001, ∗∗∗∗p < 0.0001 indicates statistical differences. Scale bars = 100 μm.

Critical to the experimental framework was ascertaining the effect of HPL supplementation on a rat NP cell population (Fig. 2). Both a 5 and 10% HPL concentration significantly increased aggrecan protein expression (Fig. 2A and B, p = 0.011 and 0.039, respectively), with collagen type II similarly being upregulated at the 10% dose (Fig. 2C and D, p = 0.017). The addition of HPL at all concentrations increased cell number, with the strongest effect observed at 10% (Fig. 2E and F, p = <0.0001). A trend towards increased cell number was also observed between 2.5% and 5% HPL (p = 0.0862). Therefore, 10% HPL supplementation was selected for subsequent experiments. Successful over-expression of miRNA-149-5p was confirmed by RT-qPCR post-transfection with a significant increase compared to the NT control (Fig. 2G, p < 0.0001). The silencing of miRNA-221-3p was also demonstrated, with its expression significantly downregulated compared to the control (Fig. 2H, p < 0.0001). Quantification of key GFs in HPL was performed by ELISA (Supplementary Fig. S1) and compared with FBS, the most commonly used serum supplement. Significantly higher levels of TGF-β (p < 0.001), VEGF (p = 0.015), and PDGF (p = 0.033) were detected in HPL, confirming its rich GF composition.

### 3.2 Dual-miRNA transfection combined with HPL supplementation increased protein expression of matrix markers while maintaining an anti-catabolic effect in rat monolayer culture

**Fig. 3:**
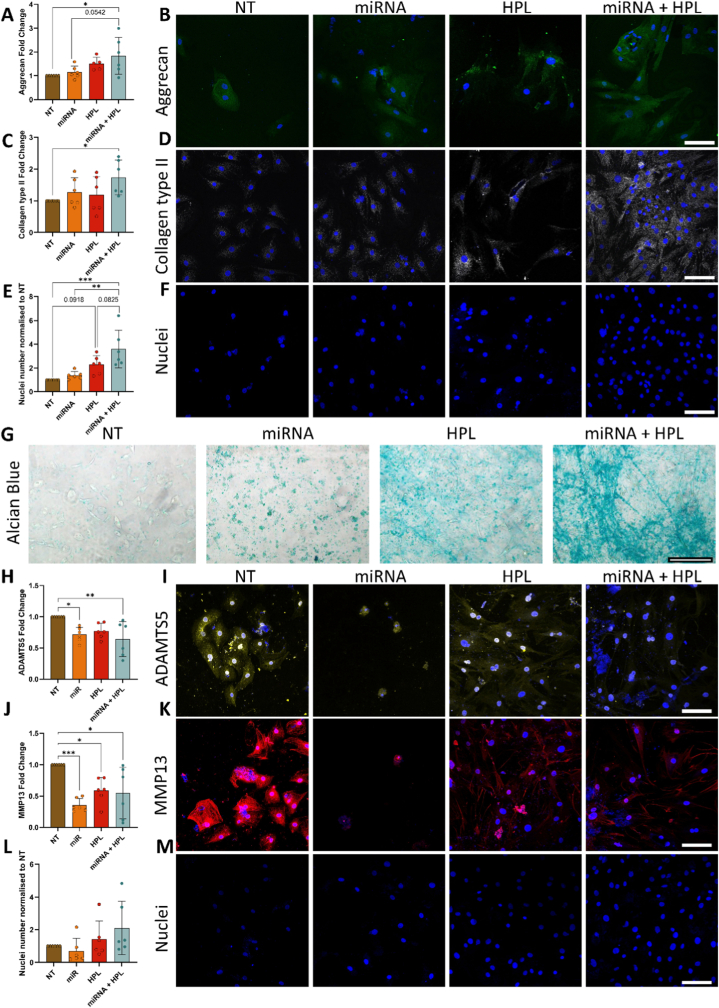
**Examination of extracellular matrix (ECM) discogenic and degrading factors in rat nucleus pulposus monolayer cultures demonstrated enhanced expression of regenerative proteins and downregulation of catabolic markers.** ECM markers assessed using immunofluorescence staining with representative images for (A & B) aggrecan (green) and (C & D) collagen type II (grey). (E & F) The number of nuclei were assessed through DAPI staining. (G) Representative images of Alcian blue staining evaluated the total accumulation of sGAGs. (H & I) Catabolic protein expression post-cytokine challenge for ADAMTS5 (yellow) and (J & K) MMP13 (red), alongside (L & M) nuclei number post-cytokine challenge. N = 6 independent biological donors, data normalised to 0% HPL control.∗p < 0.05, ∗∗p < 0.01, ∗∗∗p < 0.001 indicates statistical differences. Scale bars = 100 μm.

Upon establishing the most efficient HPL dose, the incorporation of our therapeutic dual-miRNA and the impact upon ECM protein markers was explored (Fig. 2). ECM production was enhanced in the miRNA + HPL group, with aggrecan protein levels significantly increased compared to the NT control (Fig. 3A and B, p = 0.017). Production of collagen type II also increased in the miRNA + HPL group (Fig. 3C and D, p = 0.049). Furthermore, cell number increased over 3-fold following dual-miRNA transfection combined with HPL supplementation (Fig. 3E and F, p = 0.003 vs NT, p = 0.002 vs miRNA only). A trend towards increased cell number was observed post-HPL delivery (Fig. 3E and F, p = 0.0918 vs. NT) as well as when comparing miRNA + HPL vs HPL treatment (Fig. 3E and F, p = 0.0825).Alcian blue staining of sGAGs supported these findings, with deeper staining evident in the miRNA + HPL group compared to all other treatments and NT control (Fig. 3G).

Targeting the catabolic milieu is essential for tackling IVD degeneration. Therefore, the protein expression of two frequently upregulated degradative enzymes, ADAMTS5 and MMP13, were assessed following cytokine challenge. ADAMTS5 was significantly downregulated in the dual-miRNA group (Fig. 3H and I, p = 0.031 vs NT), with the combination of HPL further bolstering this effect (p = 0.005 vs NT). MMP13 expression was significantly reduced across all treatments, with miRNA alone producing the strongest effect when compared to the control (Fig. 3J and K, p = 0.0007). HPL supplementation exerted a comparable effect, suppressing MMP13 expression (p = 0.032 for HPL only and p = 0.016 for miRNA + HPL in relation to NT). No significant changes in cell number were observed, possibly due to the cytokine challenge (Fig. 3L and M). However, an upward trend similar to the non-challenged cytokine group was observed.

### 3.3 Dual-miRNA combined with HPL enhanced production of matrix constituents in a rat ex vivo organ culture model

The effects of miRNA and HPL treatment were subsequently evaluated in a physiologically relevant microenvironment using mildly degenerated rat ex vivo disc organs. NP cell viability was preserved throughout the culture period, as determined by live/dead staining, with no significant changes observed between treatment groups (Supplementary Fig S2 A & B). This was further corroborated biochemically, with no marked changes evident in DNA content between the groups, indicating the suitability of the ex vivo model (Supplementary Fig S2 C). Histological analysis revealed that cABC treatment visibly disrupted the NP matrix, with tears evident in all groups compared to the intact Healthy control (Supplementary Fig S2 D). Due to its viscosity, the potential interference of HPL with miRNA uptake was assessed. The viscosity of HPL did not impede miRNA delivery as demonstrated by the uptake of fluorescently labelled miRDy547, which was unchanged between miRDy547 only and miRDy547 + HPL, and which remained localised within the cells following 7 days in culture (Supplementary Fig S2 E & F).

**Fig. 4:**
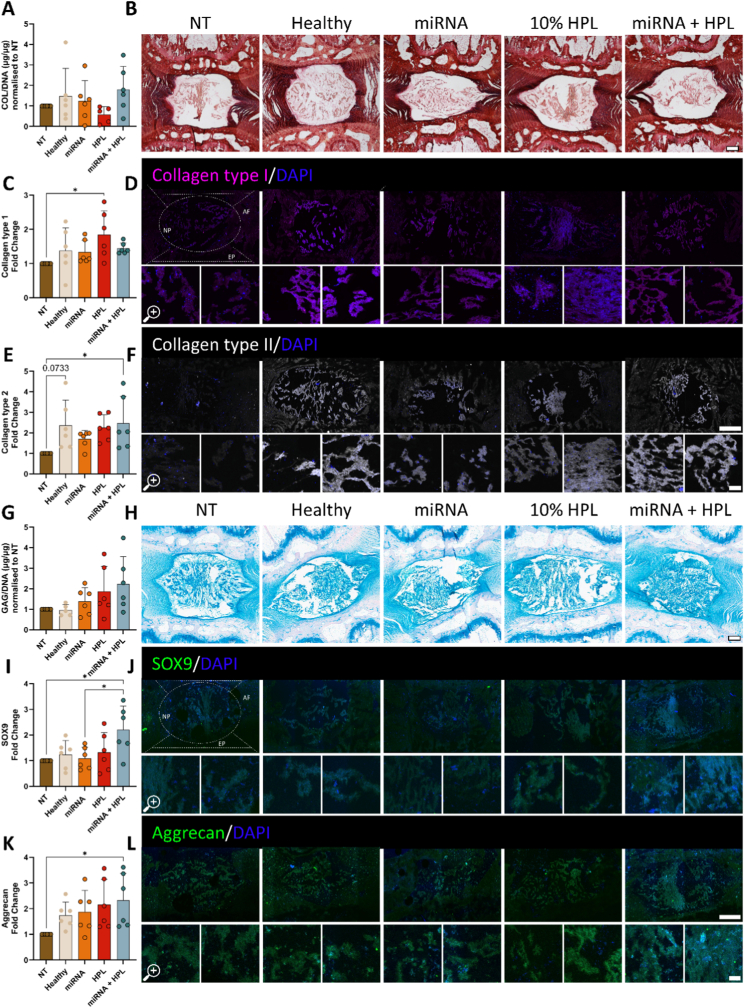
**Biochemical and histological evaluation in a rat ex vivo organ culture model of mild disc degeneration demonstrated upregulation of key extracellular matrix (ECM) constituents.** (A) Collagen content was measured biochemically and normalised to DNA content and non-treated (NT) control, with (B) Picrosirius red staining. (C & D) Specific markers were quantified using immunofluorescence, displayed alongside representative images for collagen type I (magenta) and (E & F) collagen type II (grey). (G) sGAGs were investigated quantitatively via a biochemical assay and qualitatively with (H) Alcian blue staining. (I & J) SOX9 (green) an early-stage discogenic marker and (K & L) aggrecan (green) a later-stage matrix marker were evaluated by antibody staining. N = 6 independent biological donors, data normalised to NT control. ∗p < 0.05 indicates statistical differences. Histology scale bars = 500 μm, main immunofluorescence images = 400 μm, magnified image = 50 μm. Abbreviations: NP, nucleus pulposus; AF, annulus fibrosus; CEP, cartilage endplate.

To assess the potential of our treatments in promoting ECM components, total collagen and specific collagen markers were analysed. Overall collagen levels in the NP remained unchanged following treatments (Fig. 4A and B). Collagen type I, associated with a fibrotic phenotype, was significantly upregulated in the 10% HPL only group (Fig. 4C and D, p = 0.028). A trend towards increased collagen type II expression was observed in the Healthy group compared to the NT control (Fig. 4E and F, p = 0.073). Consistent with findings from monolayer cultures, collagen type II was markedly increased following miRNA + HPL treatment relative to the NT control (Fig. 4E and F, p = 0.047).

Biochemical evaluation revealed a non-significant trend in increasing sGAG levels following miRNA + HPL delivery (Fig. 4G and H). To further explore this, key early- and late-stage proteins in the ECM were measured. SRY-box transcription factor 9 (SOX9) protein expression was significantly upregulated 2-fold by miRNA + HPL treatment relative to the degenerated control (Fig. 4I & J, p = 0.019). Consistent with monolayer findings, the later-stage matrix-related protein aggrecan was also significantly enhanced by miRNA + HPL treatment (Fig. 4K & L, p = 0.048 vs NT), suggesting a shift towards sGAG-rich matrix production. To ensure sGAGs were not leaching from the ex vivo organs, media was collected at each feed following treatment delivery. Assessment of sGAG released into the culture media at each timepoint revealed no significant differences between groups, indicating that sGAGs were retained within the NP region (Supplementary Fig S3 A & B).

### 3.4 miRNA transfection effectively suppressed catabolic markers in an ex vivo organ culture model

**Fig. 5:**
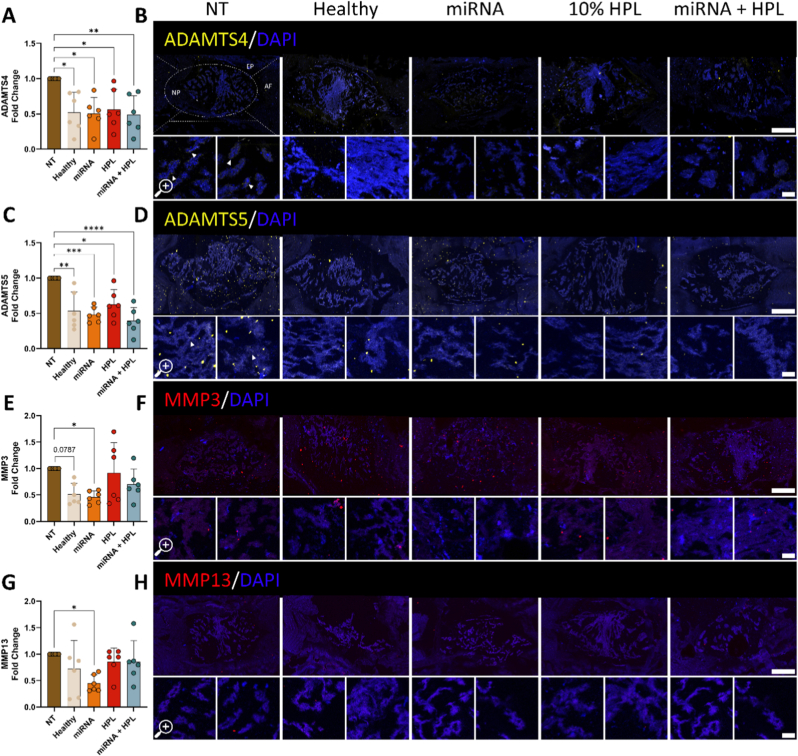
**Downregulation of protein expression of catabolic ECM degrading factors in a rat ex vivo organ culture model of mild degeneration following treatment with dual-miRNA and 10% HPL.** Representative immunofluorescence images of (A&B) ADAMTS4 (yellow), (C&D) ADAMTS5 (yellow), (E&F) MMP3 (red), and (G&H) MMP13 (red) protein expression. N = 6 independent biological donors, data normalised to NT control. ∗p < 0.05, ∗∗p < 0.01, ∗∗∗p < 0.001, ∗∗∗∗p < 0.0001 indicates statistical differences. Main image scale bar = 400 μm, magnified image = 50 μm. Abbreviations: NP, nucleus pulposus; AF, annulus fibrosus; CEP, cartilage endplate. (For interpretation of the references to colour in this figure legend, the reader is referred to the Web version of this article.)

Effective therapeutic targeting of IVD degeneration is likely to be enhanced by further reducing or dampening the catabolic milieu thereby providing a permissive environment for regeneration to occur. In this study we demonstrated clear evidence of catabolic suppression, with all treatment groups returning to levels comparable to the non-treated healthy control. All treatments significantly downregulated ADAMTS4 and ADAMTS5 protein expression when compared to the NT control, with the strongest silencing observed in the miRNA + HPL group (Fig. 5A–D, p = 0.009 for ADAMTS4, and p < 0.0001 for ADAMTS5, respectively). MMP3 expression showed an upward trend in the NT group compared to the Heathy control (Fig. 5E and F, p = 0.0787). Furthermore, miRNA treatment mirrored previous findings from our laboratory [31,32], showing a substantial reduction, over 50%, in both MMP3 and MMP13 protein expression compared to the NT control (Fig. 5E–H, p = 0.04 and p = 0.05, respectively).

### 3.5 Co-delivery of the dual-miRNA combined with HPL enhanced the expression of matrix markers in human NP cell-laden analogues

**Fig. 6:**
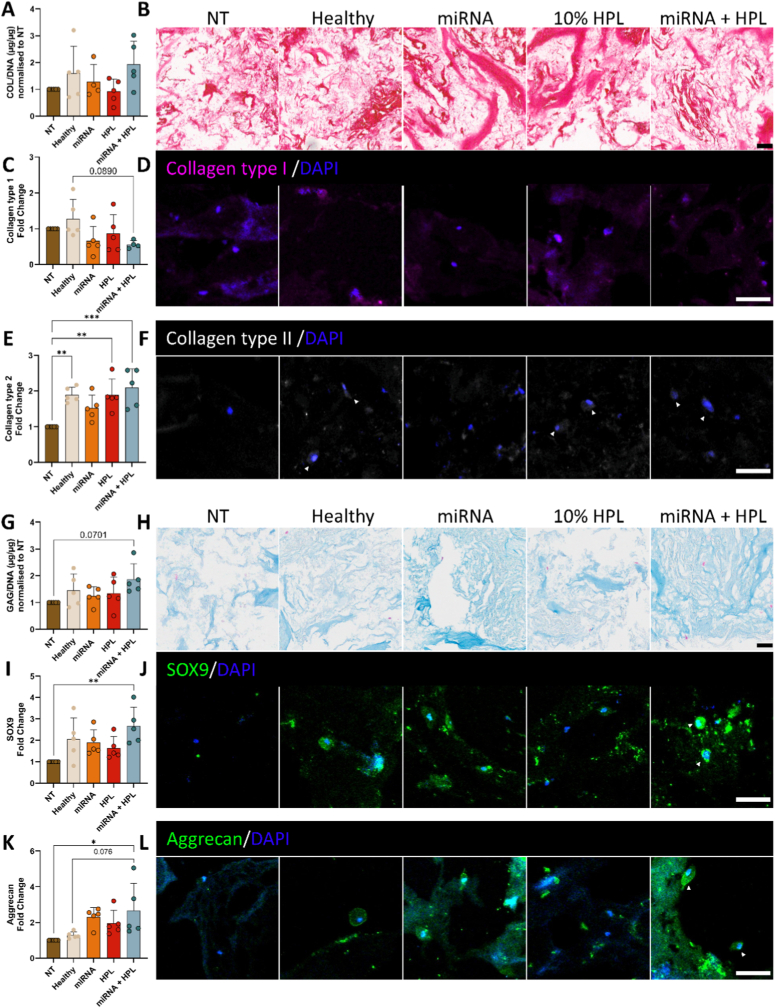
**Co-delivery of the dual-miRNA combined with HPL enhanced the expression of matrix markers in a chondroitinase ABC degenerated human cell-laden NP-ECM gel analogue.** (A) Total collagen levels were analysed biochemically and (B) histologically with picrosirius red staining. (C & D) Collagen type I (magenta) and (E & F) collagen type II (grey) protein expression was determined using immunofluorescence. (G) Total sulphated glycosaminoglycan (sGAG) content was assessed biochemically and with (H) Alcian blue staining. (I & J) Protein expression of SOX9 (green) and (K & L) aggrecan (green) was evaluated using immunofluorescence staining. N = 5 independent biological donors, all data normalised to NT control. ∗p < 0.05, ∗∗p < 0.01, ∗∗∗p < 0.001 indicates statistical differences. Representative histological images scale bar = 100 μm, representative histology staining scale bar 50 μm, immunofluorescence images scale bar = 50 μm.

Assessment of human patient-derived NP cell responses to miRNA and HPL delivery revealed promising anabolic effects. Following three weeks of culture, cell viability was comparable to that of the healthy controls across all treatment groups, with the highest viability observed in the miRNA HPL groups (p = 0.006 compared to NT control, Supplementary Fig. S4 A-D). Similarly, in the Healthy and HPL groups, cell viability was markedly higher than in the NT control (S4 A-D, p = 0.028, and p = 0.034, respectively). DNA content remained consistent across all groups with no significant differences apparent. Furthermore, no apparent differences in H&E staining were observed, confirming the biocompatibility of the model. Biochemical and histological analysis with picrosirius red staining confirmed that total collagen content did not show a significant increase by the end of the culture period (Fig. 6A and B), and collagen type I levels remained unchanged (Fig. 6C and D). However, a trend towards decreased collagen type I was observed in the miRNA + HPL group compared to the Healthy control (Fig. 6C and D, p = 0.089). In contrast, collagen type II was significantly upregulated following HPL supplementation, with a mean 1.8-fold increase compared to the NT control (p = 0.007). Treatment with miRNA + HPL further elevated collagen type II levels, resulting in a 2-fold increase (Fig. 6E–F, p = 0.0009).

Consistent with our findings in rat organ cultures, we observed an upward trend in sGAG production following miRNA + HPL treatment, as evidenced via biochemical quantification and histological staining (Fig. 6G and H, p = 0.07). Furthermore, we observed a marked increase in ECM factors with the delivery of miRNA + HPL, namely SOX9 with more than a 2.5-fold change when compared to the NT control (Fig. 6I and J, p = 0.008). miRNA + HPL treatment also enhanced aggrecan production, with an average 2.6-fold increase compared with the NT control (Fig. 6K and L, p = 0.023). A trend towards increased aggrecan expression was also observed in the miRNA + HPL treatment group compared to the Healthy control (Fig. 6K and L, p = 0.076). Overall, the expression patterns of matrix constituents closely mirrored those observed in our rat ex vivo organ culture, reinforcing the relevance of our NP-ECM analogue model as a representative ex vivo model.

### 3.6 miRNA + HPL treatment attenuated catabolic enzyme expression of human NP cells cultured in an NP cell-laden analogue

**Fig. 7:**
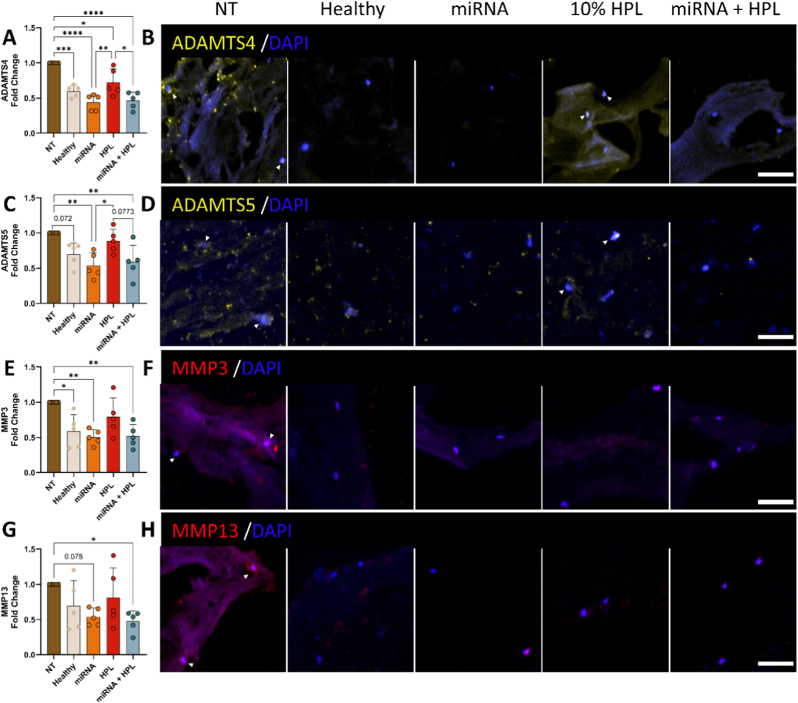
**Co-delivery of the dual-miRNA combined with HPL suppressed matrix degrading enzyme expression in a chondroitinase ABC degenerated human cell-laden NP-ECM gel analogue.** Following three weeks of culture, immunofluorescence staining was performed to measure levels of the aggrecanases (A & B) ADAMTS4 (yellow), and (C & D) ADAMTS5 (yellow), and the collagenases (E & F) MMP3 (red), and (G & H) MMP13 (red). N = 5 independent biological donors, all data normalised to NT control. ∗p < 0.05, ∗∗p < 0.01, ∗∗∗p < 0.001, ∗∗∗∗p < 0.0001 indicates statistical differences. Scale bar = 50 μm.

Modulating the response of NP cells to degeneration is essential for developing effective treatments and studies using human patient-derived cells offer valuable insights. As expected, treatment with cABC induced the highest protein levels of aggrecanases ADAMTS4 and ADAMTS5, and collagenases MMP3 and MMP13, reflecting the homeostatic imbalance characteristic of IVD degeneration. ADAMTS4 expression was reduced by over 50% compared to the NT control following either miRNA only or miRNA + HPL delivery (Fig. 7A and B, p < 0.0001 for both). Interestingly, the inclusion of miRNA-149-5p mimic and 221-3p inhibitor exerted an enhanced protective effect. ADAMTS4 expression was significantly diminished in both miRNA groups compared to HPL alone (Fig. 7A and B, p = 0.009 miRNA vs HPL, and p = 0.019 miRNA + HPL vs HPL). A similar pattern was observed for ADAMTS5, with both miRNA and miRNA + HPL treatments resulting in substantial decreases compared to the NT control (Fig. 7C and D, p = 0.002 for miRNA, and p = 0.008 for miRNA + HPL). Trends toward increased ADAMTS5 expression were observed in the NT group compared to the Healthy control (Fig. 7C and D, p = 0.072), and similarly in the HPL group compared to the miRNA + HPL group (Fig. 7C and D, p = 0.0773). Consistently, miRNA delivery produced a significant inhibitory effect compared to HPL treatment alone (Fig. 7C and D, p = 0.029). MMP3 expression was reduced by over 50% following miRNA treatment (Fig. 7E and F, p = 0.002 vs NT). Similarly, miRNA + HPL treatment significantly inhibited MMP3 in comparison to the NT control (Fig. 7E and F, p = 0.003), while HPL only treatment did not exhibit a beneficial effect. Treatment with miRNA + HPL suppressed MMP13 production compared with the NT control (Fig. 7G and H, p = 0.035), whereas miRNA only treatment showed a non-significant downward trend (Fig. 7G and H, p = 0.078). The observed alignment between the rat ex vivo organ response and patient-derived NP cells cultured in an NP analogue suggests that this system may serve as a relevant three-dimensional (3D) in vitro model for evaluating patient-specific responses to novel treatments.

## 4 Discussion

Treatment of IVD degeneration, a multifaceted condition, likely requires a multi-pronged approach, to silence degradative and inflammatory processes while promoting matrix synthesis. miRNA-based therapies have shown considerable promise, and our laboratory has identified that the combined delivery of FLR-miRNA-149-5p and a miRNA-221-3p inhibitor exerts a strong anti-catabolic effect on resident NP cells [31,32]. However, while encouraging, the observed anabolic response requires additional enhancement to achieve a meaningful regenerative outcome. Platelet lysates with their multitude of GFs, cytokines, and chemokines have demonstrated the ability to stimulate ECM deposition in vitro and improve patient outcomes in vivo [26,42]. In this study, we investigated combined dual-miRNA and HPL delivery to assess its potential as a novel therapeutic for IVD degeneration. We utilised our previously established anti-catabolic effect through FLR-miRNA-149-5p and miRNA-221-3p inhibitor delivery in both rat and human models of mild IVD degeneration. Notably, HPL supplementation promoted the expression of discogenic markers in both rat ex vivo organ culture and patient-derived cells cultured in an NP-analogue. Collectively, these findings indicate that combined dual-miRNA and HPL administration facilitates a homeostatic rebalance conducive to the formation of a restorative niche in models of IVD degeneration.

A central feature of IVD degeneration is the dysregulation and exacerbation of the inflammatory and catabolic milieu. miRNA-149-5p has demonstrated potent anti-inflammatory effects in both in vitro and in vivo studies and likely contributed to the reduced degradative enzyme response observed in this work. Qin et al. delivered miRNA-149 to NP cells in a lipopolysaccharide (LPS)-induced model of inflammation. Consistent with our findings, they reported a significant reduction in MMP3 and ADAMTS4 expression at both the gene and protein levels [43]. Chondrocytes share close morphological and functional similarities with NP cells making their treatment responses relevant to IVD research. In this context, TNF treated chondrocytes transfected with miRNA-149 rescued cell viability relative to non-treated controls, while also reducing levels of interleukin (IL)-1β, IL-6, and IL-18 in vitro [44]. In a murine OA model, miRNA-149 treatment reduced arthritis severity reflected in improved Mankin scores, and was associated with marked decreases in the inflammatory cytokines tumour necrosis factor (TNF)-α, IL-1β, and IL-6 compared with negative controls [45]. Both TNF and IL-1β are key regulators of MMP and ADAMTS expression that exacerbate IVD degeneration [[46], [47], [48], [49]]. Previous bioinformatics analysis from our laboratory of validated gene targets of miRNA-149 identified enriched pathways related to response to LPS, execution phase of apoptosis, response to peptidoglycan, and regulation of signal transduction, highlighting it's anti-catabolic effects. The inclusion of miRNA-149-5p in our therapeutic dual-miRNA may explain the suppression of degradative enzyme expression observed in our systems.

Interestingly, HPL delivery alone did not exhibit this anti-catabolic effect, requiring the incorporation of our dual-miRNA. Backly and colleagues reported that, in keratinocytes, lysate-mediated enhancement of wound healing is preceded by a transient inflammatory response accompanied by cytokine recruitment [50]. Furthermore, Pereira et al. found that chondrocytes treated with lysates exhibited an inflammatory response when cultured in the presence of IL-1α, with a more pronounced effect than IL-1α stimulation alone [51]. In our ex vivo organ and NP analogue culture systems, we observed that dual-miRNA and dual-miRNA + HPL were more effective at suppressing the expression of catabolic factors than HPL supplementation alone. This combined therapeutic effect has likewise been exploited in inflammatory bowel disease, whereby the incorporation of an MMP-9-responsive hydrogel with an anti-inflammatory drug elicited promising effects in an in vivo model of acute colitis [52]. Alongside a significantly reduced histopathology score, the joint treatment significantly reduced TNF, IL-6, IFN-γ, and MMP9, supporting multimodal strategies. In the context of IVD degeneration, expression of ADAMTS and MMP family members in the disc is closely associated with inflammatory signalling, and is stimulated by key regulators such as TNF and IL-1β [46,53]. The responses observed here with singular HPL may therefore reflect an initial inflammatory reaction triggered by HPL delivery that had not yet abated by the end of the culture period, and that was not apparent in the dual-miRNA + HPL delivery groups.

While the above studies focus on shorter timepoints, more pronounced anti-inflammatory effects may only become evident over longer study durations. Studies in cartilage, similar to the NP due to its avascular nature, biochemical composition, and mechanical load bearing environment have demonstrated this response as being shorter term. In vivo implantation of a platelet lysate hydrogel scaffold for cartilage repair in rabbits resulted in significantly higher populations of anti-inflammatory M2 macrophages compared to the inflammatory M1 macrophages up to 42 days post-implantation [54]. This has been reflected clinically in OA, with Filardo et al. reporting increased post-injection swelling and pain in lysate-treated patients compared to controls [55]. Khoshbin and colleagues likewise reported significantly more occurrences of adverse events, including post-injection swelling and pain in their lysate group over controls in the treatment of OA [56]. In both cases these events were transient, which may suggest a temporal elevated inflammatory response that was not captured in our relatively shorter culture times. The 21-day culture period allowed for direct comparison between rat ex vivo organ culture and human NP cell-laden NP-ECM analogue models; however, increasing this time period may be beneficial to assess fibrotic changes. We observed an increase in collagen type I protein expression following HPL delivery in rat ex vivo organ culture, potentially indicating fibrotic remodelling. Furthermore, longer culture periods could provide insight into the stability of the newly formed matrix post-dual-miRNA + HPL treatment. While longer culture periods are infeasible in rat ex vivo organ culture, the human NP cell-laden NP-ECM analogue may allow for extended culture durations and could be leveraged in future studies.

A key objective of this work was to enhance the effect of dual-miRNA delivery in relation to the promotion of anabolic factors. We observed a synergistic effect when combining the dual-miRNA-with HPL, resulting in greater upregulation of matrix markers than with dual-miRNA delivery alone. HPL is enriched with GFs, containing over 1400 proteins, highlighting its regenerative potential [57]. Amongst these, members of the TGF superfamily, VEGF, PDGF, fibroblastic growth factor (FGF), epidermal growth factor (EGF), and bone morphogenic proteins (BMPs) are often over-represented [[58], [59], [60]]. Similar to the promotion of matrix factors identified in this work, platelet-related treatments have shown promising regenerative effects in NP cells, with one study reporting that 2.5% platelet-rich plasma (PRP) upregulated the expression of collagen type II, aggrecan, and SOX9 in rat NP cells [61]. A recent study demonstrated significant increases in aggrecan and collagen type II protein expression following pure PRP treatment in IL-1β challenged rat NP cells, consistent with the findings presented here [62]. Likewise, in a porcine ex vivo organ culture model, delivery of PRP resulted in significantly increased GAGs when compared to the degenerated control [63]. In this study, HPL was utilised in place of PRP, although both have demonstrated clinical utility. The use of GMP-grade manufacturing and a single batch throughout ensured a high degree of consistency and standardisation in HPL composition, which is particularly important given the incorporation of the dual-miRNA. In addition, HPL has been reported to exert a more pronounced immunomodulatory effect in vivo, an important consideration given the catabolic milieu associated with IVD degeneration [64] While both PRP and HPL have demonstrated therapeutic benefit in knee osteoarthritis (KOA) models, HPL appears to yield superior clinical outcomes [65,66]. This may be attributable to its higher concentration of bioactive growth factors [67], which has been associated with greater improvements in range of motion, visual analogue scale (VAS) scores, and Western Ontario and McMaster Universities Osteoarthritis Index (WOMAC) scores. In the present work, the inclusion of a GF cocktail HPL combined with the immunomodulatory effects of our dual-miRNA likely promoted the upregulation of key matrix associated markers including aggrecan, SOX9, and collagen type II.

Inhibition of miRNA-221 has been associated with enhanced matrix marker expression, as demonstrated in a recent study where stem cell-laden, miRNA-221 activated hydrogels showed significant increases in sGAG and collagen type II levels after 21 days of chondrogenic culture [68]. miRNA-221 has been shown to directly target and downregulate tissue inhibitor of metalloproteinase-2 (TIMP-2), a key regulator of the MMP family, leading to amplified MMP expression in degenerative conditions. However, silencing miRNA-221 in fibroblastic cells derived from the spinal ligamentum flavum restored TIMP-2 expression and corrected aberrant ECM degradation enabling the resumption of aggrecan and collagen type II production [69]. In this study, similar patterns were observed, although some positive effects were noted when either the dual-miRNA or HPL was delivered alone, the strongest effects occurred when they were combined. Consistent with previous studies, we observed significant increases in collagen type II protein expression following treatment with dual-miRNA and HPL. Additionally, both rat and human culture systems showed an upward trend in sGAG production, along with significant increases to SOX9 and aggrecan, key precursors of the mature protein. We have previously reported bioinformatics analysis of validated gene targets and enriched pathways by miRNA-221 [32]. Identified target genes were enriched for matrix-related processes, including MMP2, TIMP-3, both of which play central roles in matrix remodelling [4]. Furthermore, C-X-C Motif Chemokine Ligand 12 (CXCL12) was significantly enriched, a factor linked to inflammation-induced apoptosis and ECM disruption [70]. Collectively, this strengthens the mechanistic role of miRNA-221-3p as a part of our therapeutic dual-miRNA. Taken together, these findings suggest a synergistic effect whereby co-delivery of dual-miRNA and HPL enhances matrix marker expression more effectively than either treatment alone.

In addition to investigating the combined delivery of a therapeutic dual-miRNA and HPL, we developed a biomimetic ECM 3D culture platform to study potential therapeutic targets using human cells. In vitro systems that closely replicate physiological conditions are crucial to gain a deeper understanding of cellular responses to treatments. NP cells exhibit differential expression of discogenic markers depending on whether they are cultured in 2D or 3D systems, with 3D culture promoting increased expression of NP-like markers, indicative of a more representative phenotype [71,72]. To better represent physiological conditions, we employed a decellularised NP-ECM matrix mimicking the matrix composition of a mildly degenerated Pfirrmann Grade 3 disc as our culture system for human NP cells, allowing us to model the structural environment of a degenerated matrix.

While decellularised matrices or biologically similar constituents have shown promise as therapeutics in the IVD field [13,[73], [74], [75], [76]], in this work we demonstrate the versatility of using a decellularised NP-ECM matrix as an in vitro biomimetic culture environment. Complementary to our work with human cell-laden NP-ECM gel analogues, Yuan and colleagues reported that fibroblasts seeded atop rabbit-derived NP matrix exhibited significantly enhanced collagen type II expression. Furthermore, they observed a strong reduction in collagen type I expression, suggesting a shift away from the fibroblastic phenotype towards more NP-like behaviour [77]. Rashidi and colleagues demonstrated differences in NP cell morphology and surface marker expression in biomimetic age-related collagen-proteoglycan gels [78]. Cells cultured in gels resembling aged matrix demonstrated increased integrin-beta-1 (ITGB1), indicative of degenerated discs [79]. A matrix microenvironment characteristic of a mildly degenerated disc was employed here, however the cellular response to an environment representing a more severely degenerated disc could be explored through alteration of the ECM and CS content.

Peng et al. employed bone marrow derived stem cells (BMSCs) seeded on decellularised NP gels, whereby they identified significant upregulation of protein expression of collagen type II, aggrecan, and the NP markers CD24, and keratin 19 (KRT19) when cultured in 3D, suggesting that the NP gels were supporting a pro-regenerative NP-like response [80]. Collectively, these studies, together with our own, highlight the importance of implementing physiologically relevant culture systems, as they advance our understanding of cellular response to degenerative matrix alterations, while simultaneously providing a platform for the effective evaluation and optimisation of emerging therapeutics. Such tuneable environments have been successfully utilised in the generation of robust in vitro models for the development of intestinal organoids, whereby altering the mechanics of the hydrogel system imparts different cues and therefore developmental characteristics [81]. Our culture system not only provided a tuneable, viable 3D ECM niche for cell encapsulation, mimicking degenerated NP tissue, but also demonstrated its utility as an in vitro platform for testing therapeutics.

Although no adverse effects on human NP cells viability or matrix synthesis were observed in the NP-ECM gel analogue, the potential for immunogenic response or species-specific matrix effects should be considered when using xenogeneic bovine ECM. Reconstituted bovine collagen has been reported to elicit an allergic reaction in 3% of the population [82], reinforcing the importance of effictive decellularisation. Importantly, previous studies using similarly decellularised bovine ECM, combined with porcine nasal chondrocytes demonstrated sustained cell viability over 14 days of culture and sGAG production, indicating no detrimental cellular effects [75]. Furthermore, decellularised bovine ECM hydrogels encapsulating human NP cells and MSCs have shown enhanced viability over 21 days in culture compared to controls, along with evidence of newly synthesised matrix [83]. Collectively, these findings, together with our results, support the use of xenogeneic decellularised matrices as cytocompatible platforms for cell encapsulation. However, it is important to recognise that while decellularisation reduces cellular antigenicity, residual species-specific epitopes may persist within xenogeneic ECM, potentially eliciting immune responses and influencing cell behaviour. This consideration is particularly relevant for in vivo applications, as opposed to in vitro culture systems.

Importantly, our results between ex vivo organ culture and NP-ECM-analogue systems were found to be comparable, highlighting their utility as ex vivo and in vitro models of intervertebral disc degeneration. Ex vivo organ culture represents one of the most physiologically relevant systems available for pre-clinical evaluation of developing treatments. Although straightforward to implement, the provision of an ECM niche for cells may be valuable for interrogating cellular responses to a range of potential treatments. Furthermore, it provides a 3D culture system that can be populated with patient-derived cells, thereby enhancing the translatability of prospective therapeutics. The employment of viable ex vivo and in vitro models for screening of therapies is highly relevant and in keeping with the 3R principles of replacement, reduction, and refinement of animal numbers being used in scientific research [84,85].

cABC is well established as a potent enzyme for promoting sGAGs cleavage, while also inducing the upregulation of matrix-degrading proteins in ex vivo studies and large animal models [[86], [87], [88]]. cABC degeneration has been correlated with biomechanical changes associated in rat IVDs, primarily due to a loss in sGAGs [89]. However, the mild degeneration induced here did not result in a significant reduction in sGAGs sufficient to cause measurable biomechanical changes. The inclusion of a cABC challenge induces a catabolic response indicative of the degenerated disc microenvironment. This was modelled here by the upregulation of members of the ADAMTS and MMP families, supporting its use as a model of mild degeneration to evaluate strategies aimed at suppressing catabolism and promoting matrix synthesis. Finally, the system is tuneable, with ECM and CS composition adjustable to model different grades of degeneration, allowing greater exploratory capability.

The delivery of the dual-miRNA and HPL resulted in no cytotoxic effects in either culture model, supporting the safety of allogeneic HPL in the IVD field. Cell viability was unchanged in the rat ex vivo organ culture model and increased in the human NP cell-laden NP-ECM analogue following HPL and miRNA + HPL treatment. A recent study delivering allogeneic platelet lysate to degenerated rabbit IVDs similarly reported no adverse effects on cell viability [90]. Furthermore, sirtuin 1 (SIRT1) was upregulated, a factor linked to enhanced resistance to oxidative stress, suggesting an immuno-protective effect. Given the immune-privileged nature of the IVD, allogeneic HPL may represent a therapeutically relevant strategy, however, further studies are required to fully interrogate potential immunogenicity. Previous work from our laboratory has similarly identified no adverse effects from the delivery of FLR-dual-miRNA complexes to rat ex vivo organ culture [31,32], and the FLR complex has been safely delivered in vivo [91,92], highlighting the potential of this combinatorial treatment in targeting IVD degeneration.

Notwithstanding the strengths of the approaches employed in this work, several limitations warrant consideration. Confirmation of miRNA-149-5p overexpression and miRNA-221-3p silencing by RT-qPCR was confined to monolayer cultures, additional studies would benefit from quantifying expression levels in both ex vivo organ culture and the cell-laden NP-ECM analogues. However, delivery of our dual-miRNA in both ex vivo and cell-laden NP-ECM analogues did confer functional outcomes, as evidenced by its uptake and effect. Catabolic enzyme expression was evaluated semi-quantitatively through staining; however more robust conclusions could be drawn from quantitative assessment of pro-inflammatory cytokines. Unfortunately, this was not feasible with the current culture set-up for both rat and human models, as ELISA readouts fell below the measurable assay range. Measurements from human discectomy tissue are typically in the pg/mL level for factors such as TNF and IL-1β [[93], [94], [95]]. To achieve these measurable levels would likely require either non-physiological cell numbers or an artificially high inflammatory stimulus, neither of which reflects the human IVD or would be relevant for translating the combined dual-miRNA + HPL treatment. The use of high sensitivity ELISAs may also aid in measuring cytokines, as perhaps would earlier timepoints in the culture period. Further evaluation of protein expression using complementary techniques such as Western blotting would offer additional validation to the work. However, the culture methods employed in this study, particularly the ex vivo organ culture model, are inherently limited by low cell numbers, approximately 3-4 x 10^4^ cells per rat IVD [96], which poses challenges for such techniques. Nevertheless, the consistent cellular responses observed across both the ex vivo organ culture and cell-laden NP-ECM analogue suggest that future studies could scale up the model to generate sufficient material for protein expression analyses, including Western blotting. This study included a limited number of human donors which prevents stratification by Pfirrmann grade but is sufficient for this exploratory work. We also used ex vivo organ culture and a human cell-laden NP-ECM gel analogue model, both of which lack in vivo tissue interactions but are useful for studying cellular responses to potential treatments.

In summary, we evaluated a novel dual FLR-miRNA and HPL therapy for the treatment of mild IVD degeneration across multiple species and culture systems. The therapy demonstrated pro-regenerative and anti-catabolic effects in both rat and human NP models, highlighting its potential to suppress matrix degradation while promoting matrix marker expression. Alongside this, we developed a tuneable human cell-laden NP-ECM gel analogue culture system representative of the microenvironmental ECM niche typically found within the IVD, which showed responses comparable to ex vivo organ culture. Together, these findings offer a promising avenue for the treatment of low back pain.

## 5 Conclusion

Development of injectable therapeutics to treat degeneration of the IVD is a challenging endeavour, requiring the suppression of catabolic factors concurrent to restoration of the degenerated ECM. In this study, FLR-miRNA-149-5p mimic and 221-3p inhibitor combined with HPL restored homeostatic balance in rat and human NP cells through suppression of matrix degrading factors implicated in progressive IVD deterioration (MMP and ADAMTS families). Alongside this, we demonstrated promotion of regenerative marker expression, factors known to be required for the rebuilding of the ECM (SOX9, aggrecan, and collagen type II). Using a tuneable human culture model system that recapitulates a mildly degenerated disc microenvironment, we observed responses consistent with ex vivo animal derived organ cultures, supporting the translational relevance of this approach. Collectively, these findings highlight a promising novel approach to target early-stage IVD degeneration and low back pain through a single injectable therapy modulating the expression of key markers in the degenerative cascade.

## Funding

This project has received funding from the 10.13039/501100000781European Research Council (ERC) under the European Union's Horizon 2020 research and innovation program (grant agreement ERC-2019-CoG-864104; INTEGRATE).

## CRediT authorship contribution statement

**Tara Ní Néill:** Conceptualization, Data curation, Formal analysis, Investigation, Methodology, Software, Validation, Visualization, Writing – original draft, Writing – review & editing. **Niamh Wilson:** Data curation, Formal analysis, Methodology, Writing – review & editing. **Jijo Thomas:** Data curation, Formal analysis, Methodology, Writing – review & editing. **Jake McDonnell:** Data curation, Formal analysis, Methodology, Writing – review & editing. **Stacey L. Darwish:** Investigation, Resources, Writing – review & editing. **Joseph S. Butler:** Investigation, Resources, Writing – review & editing. **Fergal J. O'Brien:** Writing – review & editing. **James E. Dixon:** Resources, Writing – original draft. **Caroline M. Curtin:** Writing – review & editing. **Conor T. Buckley:** Conceptualization, Data curation, Formal analysis, Funding acquisition, Investigation, Methodology, Project administration, Resources, Software, Supervision, Validation, Visualization, Writing – review & editing.

## Declaration of competing interest

The authors declare that they have no known competing financial interests or personal relationships that could have appeared to influence the work reported in this paper.

## Data Availability

Data will be made available on request.
