# Supplementary material for: Restoring disc matrix homeostasis: Dual-miRNA and human platelet lysate as a novel therapeutic strategy
Source: Mater Today Bio. 2026 May 3;38:103190. doi: 10.1016/j.mtbio.2026.103190 (PMC13153650; doi:10.1016/j.mtbio.2026.103190)
Supplement: Multimedia component 1 [file mmc1.docx]

Supplementary materials

All materials related to this research will be made available at 10.5281/zenodo.18786635


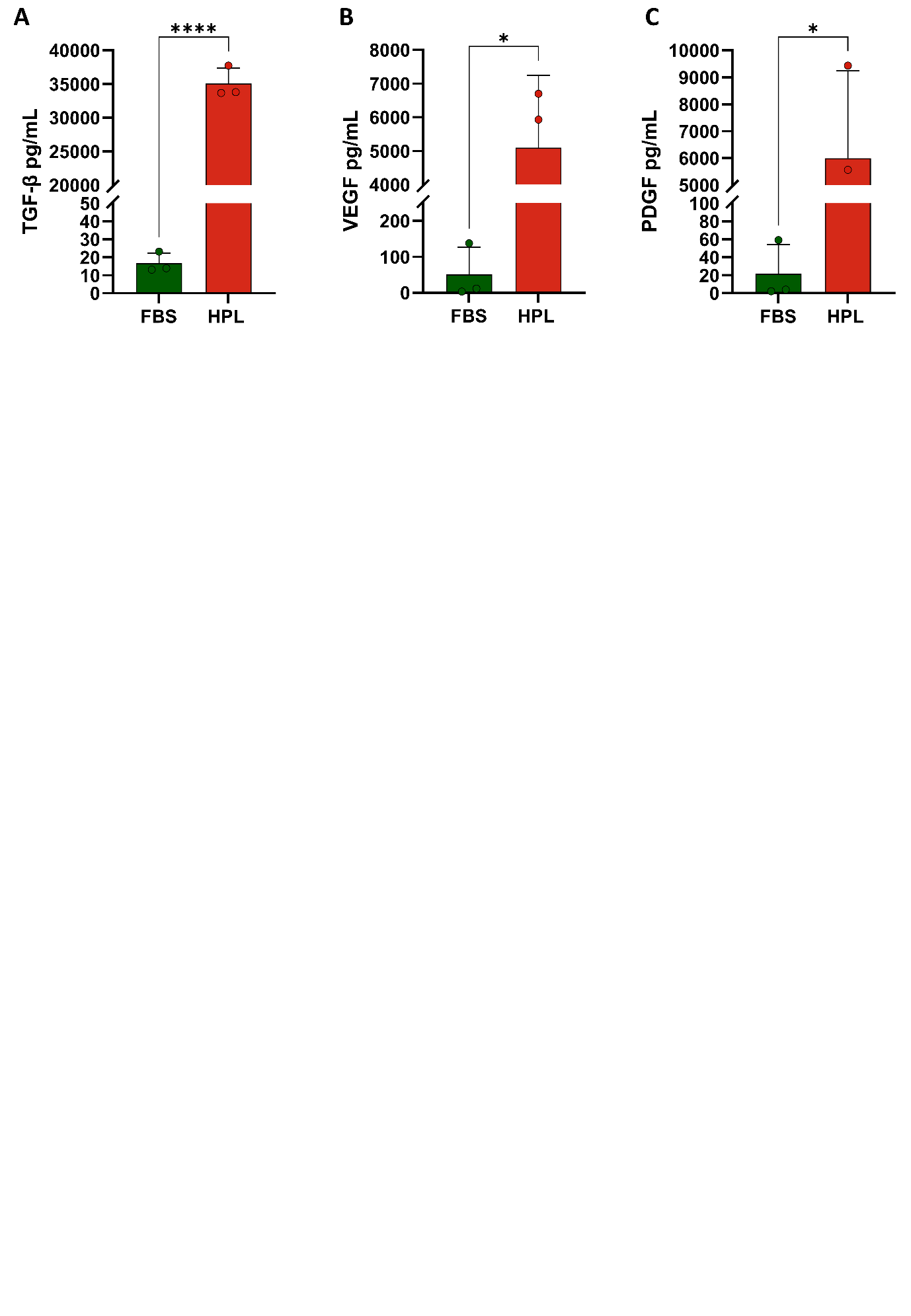


**Supplementary Figure S1. Quantification of key growth factors in human platelet lysate (HPL) compared with foetal bovine serum (FBS). A.** Transforming growth factor-β (TGF-β), **B.** Vascular endothelial growth factor (VEGF)**, C.** Platelet-derived growth factor (PDGF). N = 3 technical replicates from the same batch employed throughout the experiments presented in this work. * p < 0.05, **** p < 0.0001


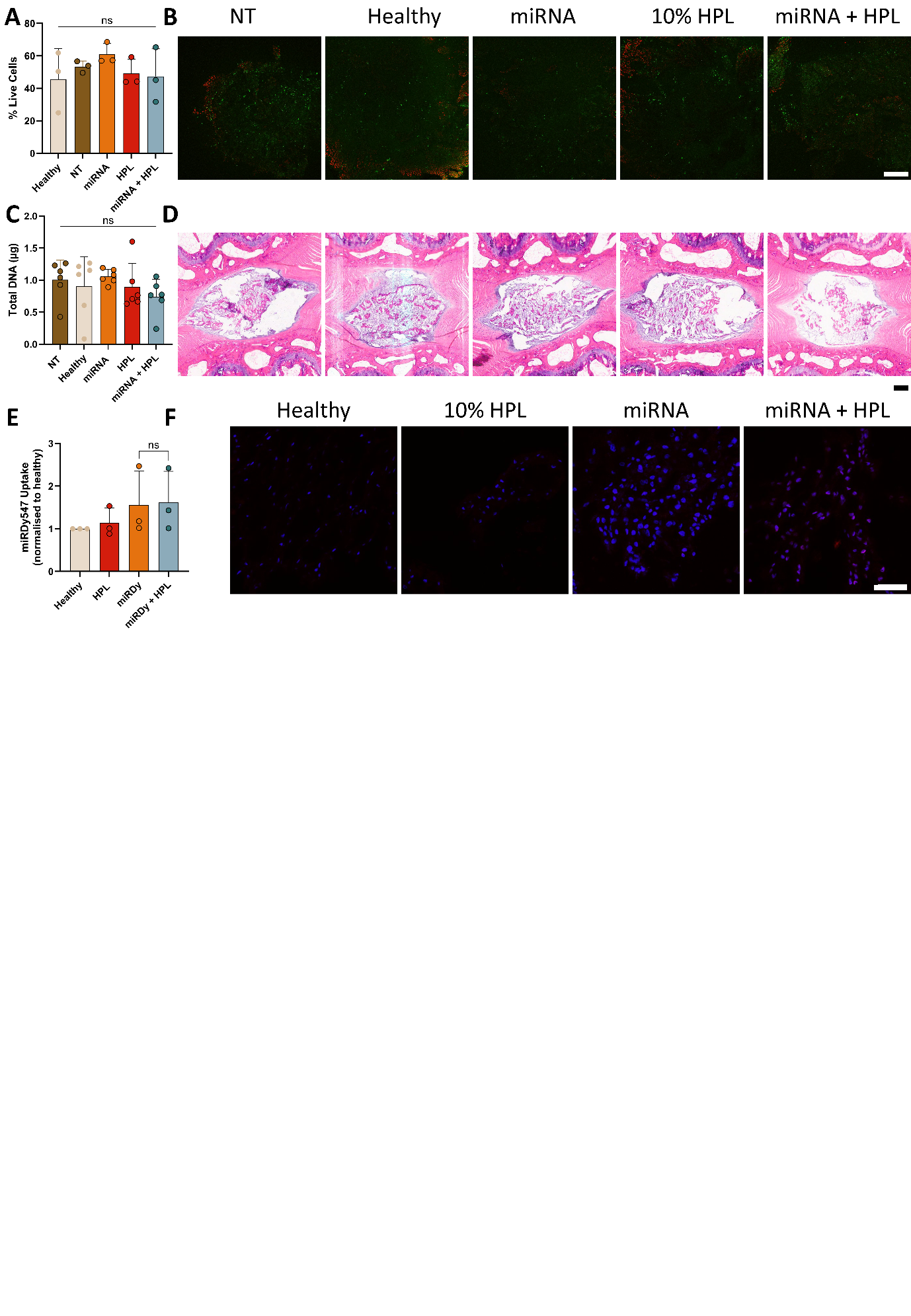


**Supplementary Figure S2. Cell viability and miRNA uptake in rat ex vivo organ cultures. (A)** Quantified cell viability (%) in organ cultures as assessed by **(B)** live/dead staining following 21 days of culture. (**C)** Total DNA was quantified biochemically and cellular morphology by evaluated using (**D)** haematoxylin and eosin staining. (**E** & **F)** Uptake of the fluorescently labelled miRDy547 was measured after 7 days of culture, in isolation or with the supplementation of 10% HPL. N = ≥ 3 independent biological donors. Live/dead scale bar = 200 µm, histology scale bar = 500 µm, miRDy547 uptake scale bar = 100 µm, ns = non-significant.

**
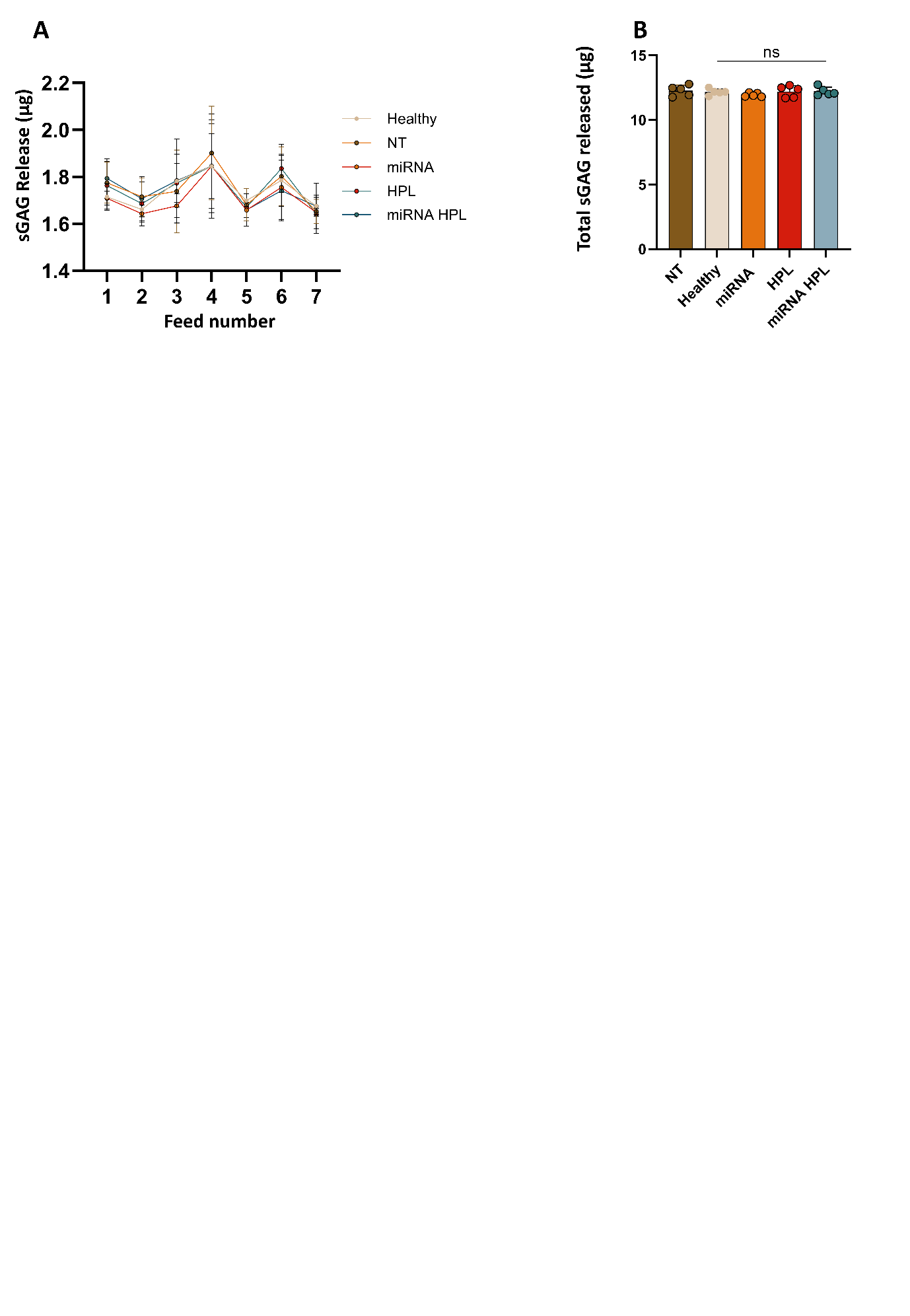
Supplementary Figure S3. Sulphated glycosaminoglycans (sGAGs) released into the media during rat ex vivo organ cultures. (A)** sGAG levels at each feeding showed no significant differences and (**B)** Total sGAG release was also not significantly different following 21 days of culture. N = 5 independent biological donors, ns = non-significant.


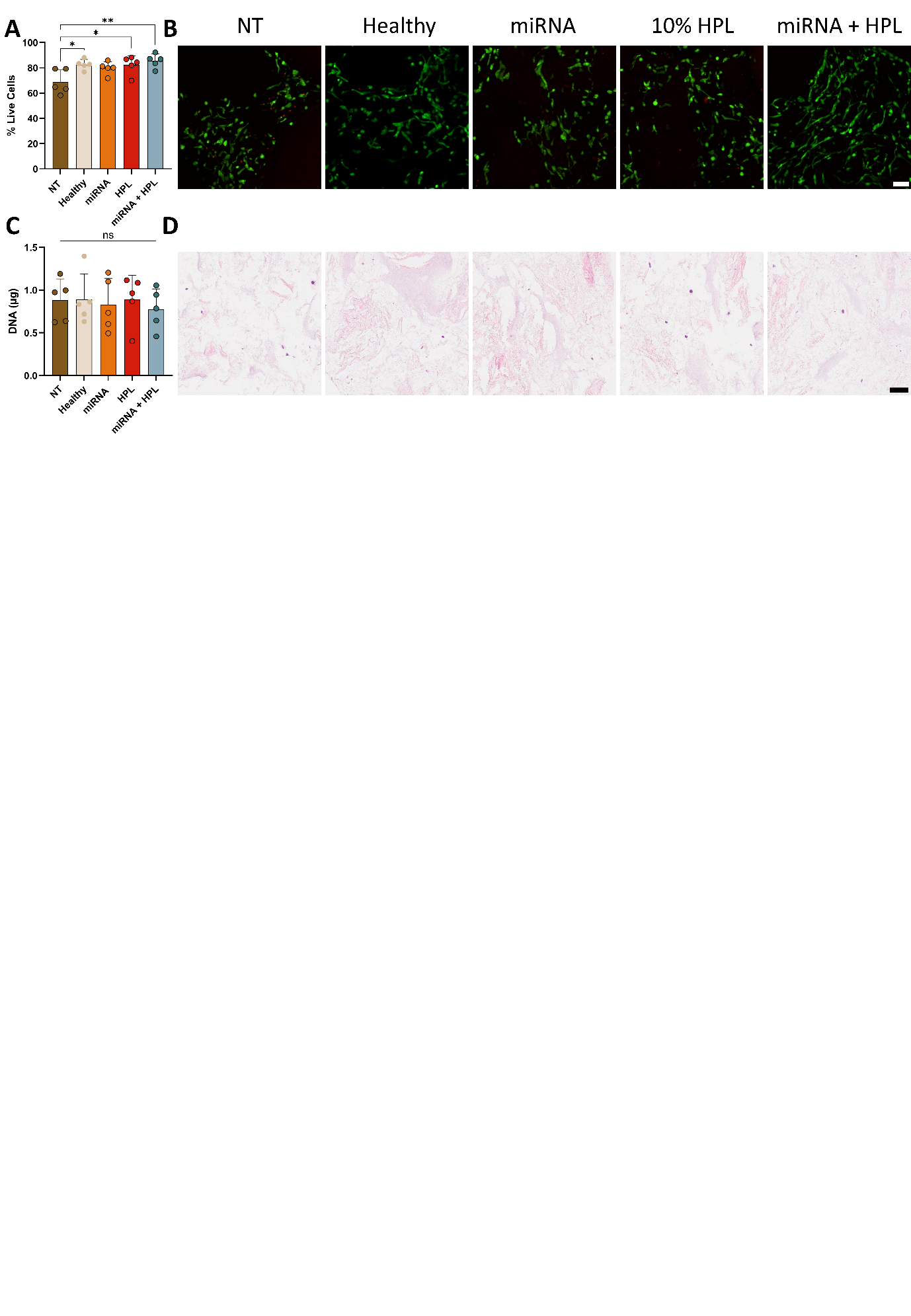


**Supplementary Figure S4. Cell viability and proliferation of human cell-laden NP-ECM gel analogues following 21 days of culture .**  (A & B) Viability was quantified with live/dead staining, (C & D) DNA content and representative haematoxylin and eosin staining. * p < 0.05, ** p < 0.01 indicates statistical differences. N = 5 independent biological donors. Scale bar = 100 µm.

Supplementary Table 1: Listing of all p-values greater than 0.1 for every group comparison conducted in this study. This table includes all non-significant differences observed across experimental conditions, providing a complete overview of comparisons that did not reach statistical significance.

| Figure | Comparison | p-value |
| --- | --- | --- |
| 2A | 0% vs. 2.5% | 0.5025 |
| 2A | 2.5% vs. 5% | 0.1859 |
| 2A | 2.5% vs. 10% | 0.4554 |
| 2A | 5% vs. 10% | 0.9300 |
| 2C | 0% vs. 2.5% | 0.6971 |
| 2C | 0% vs. 5% | 0.1433 |
| 2C | 2.5% vs. 5% | 0.6586 |
| 2C | 2.5% vs. 10% | 0.1498 |
| 2C | 5% vs, 10% | 0.7116 |
| 2E | 5% vs. 10% | 0.8352 |
| 3A | NT vs. miRNA | 0.9315 |
| 3A | NT vs. HPL | 0.2518 |
| 3A | miRNA vs. HPL | 0.5422 |
| 3A | HPL vs. miRNA HPL | 0.6057 |
| 3C | NT vs. miRNA | 0.7405 |
| 3C | NT vs. HPL | 0.8845 |
| 3C | miRNA vs. HPL | 0.9912 |
| 3C | miRNA vs. miRNA HPL | 0.3111 |
| 3C | HPL vs. miRNA HPL | 0.1965 |
| 3E | NT vs. miRNA | 0.8763 |
| 3E | miRNA vs. HPL | 0.3282 |
| 3H | NT vs. HPL | 0.1050 |
| 3H | miRNA vs. HPL | 0.9284 |
| 3H | miRNA vs. miRNA HPL | 0.8639 |
| 3H | HPL vs. miRNA HPL | 0.5227 |
| 3J | miRNA vs. HPL | 0.3373 |
| 3J | miRNA vs. miRNA HPL | 0.5083 |
| 3J | HPL vs. miRNA HPL | 0.9888 |
| 3L | NT vs. miRNA | 0.9618 |
| 3L | NT vs. HPL | 0.9053 |
| 3L | NT vs. miRNA HPL | 0.3017 |
| 3L | miRNA vs. HPL | 0.6587 |
| 3L | miRNA vs. miRNA HPL | 0.1351 |
| 3L | HPL vs. miRNA HPL | 0.6778 |
| 4A | Healthy vs. NT | 0.8907 |
| 4A | Healthy vs. miRNA | 0.9898 |
| 4A | Healthy vs. HPL | 0.5003 |
| 4A | Healthy vs. miRNA HPL | 0.9728 |
| 4A | NT vs. miRNA | 0.9914 |
| 4A | NT vs. HPL | 0.9416 |
| 4A | NT vs. miRNA HPL | 0.5634 |
| 4A | miRNA vs. HPL | 0.7647 |
| 4A | miRNA vs. miRNA HPL | 0.8196 |
| 4A | HPL vs miRNA HPL | 0.2150 |
| 4C | Healthy vs. NT | 0.6205 |
| 4C | Healthy vs. miRNA | 0.9999 |
| 4C | Healthy vs. HPL | 0.4082 |
| 4C | Healthy vs. miRNA + HPL | 0.9991 |
| 4C | NT vs. miRNA | 0.7085 |
| 4C | NT vs. miRNA + HPL | 0.4697 |
| 4C | miRNA  vs. HPL | 0.3315 |
| 4C | miRNA  vs. miRNA + HPL | 0.9945 |
| 4C | HPL vs. miRNA + HPL | 0.5543 |
| 4E | Healthy vs. miRNA | 0.6623 |
| 4E | Healthy vs. HPL | 0.9993 |
| 4E | Healthy vs. miRNA + HPL | 0.9995 |
| 4E | NT vs. miRNA | 0.6305 |
| 4E | NT vs. HPL | 0.1166 |
| 4E | miRNA  vs. HPL | 0.7936 |
| 4E | miRNA  vs. miRNA + HPL | 0.5346 |
| 4E | HPL vs. miRNA + HPL | 0.9918 |
| 4G | Healthy vs. NT | >0.9999 |
| 4G | Healthy vs. miRNA | 0.9229 |
| 4G | Healthy vs. HPL | 0.4402 |
| 4G | Healthy vs. miRNA + HPL | 0.1531 |
| 4G | NT  vs. miRNA | 0.9375 |
| 4G | NT  vs. HPL | 0.4430 |
| 4G | NT  vs. miRNA + HPL | 0.1455 |
| 4G | miRNA  vs. HPL | 0.8746 |
| 4G | miRNA  vs. miRNA + HPL | 0.4832 |
| 4G | HPL vs. miRNA + HPL | 0.9549 |
| 4I | Healthy vs. NT | 0.9603 |
| 4I | Healthy vs. miRNA | 0.9941 |
| 4I | Healthy vs. HPL | 0.9990 |
| 4I | Healthy vs. miRNA + HPL | 0.0870 |
| 4I | NT vs. miRNA | 0.9987 |
| 4I | NT vs. HPL | 0.8836 |
| 4I | miRNA  vs. HPL | 0.9638 |
| 4I | HPL vs. miRNA + HPL | 0.1429 |
| 4K | Healthy vs. NT | 0.4784 |
| 4K | Healthy vs. miRNA | 0.9983 |
| 4K | Healthy vs. HPL | 0.8837 |
| 4K | Healthy vs. miRNA + HPL | 0.6957 |
| 4K | NT vs. miRNA | 0.3209 |
| 4K | NT vs. HPL | 0.1045 |
| 4K | miRNA  vs. HPL | 0.9675 |
| 4K | miRNA  vs. miRNA + HPL | 0.8501 |
| 4K | HPL vs. miRNA + HPL | 0.9958 |
| 5A | Healthy vs. miRNA | >0.9999 |
| 5A | Healthy vs. HPL | 0.9981 |
| 5A | Healthy vs. miRNA + HPL | 0.9992 |
| 5A | miRNA  vs. HPL | 0.9920 |
| 5A | miRNA  vs. miRNA + HPL | >0.9999 |
| 5A | HPL vs. miRNA + HPL | 0.9825 |
| 5C | Healthy vs. miRNA | 0.9841 |
| 5C | Healthy vs. HPL | 0.9089 |
| 5C | Healthy vs. miRNA + HPL | 0.6371 |
| 5C | miRNA  vs. HPL | 0.6438 |
| 5C | miRNA  vs. miRNA + HPL | 0.9050 |
| 5C | HPL vs. miRNA + HPL | 0.1936 |
| 5E | Healthy vs. miRNA | 0.9975 |
| 5E | Healthy vs. HPL | 0.1970 |
| 5E | Healthy vs. miRNA + HPL | 0.8429 |
| 5E | NT vs. HPL | 0.9886 |
| 5E | NT vs. miRNA + HPL | 0.4516 |
| 5E | miRNA  vs. HPL | 0.1082 |
| 5E | miRNA  vs. miRNA + HPL | 0.6679 |
| 5E | HPL vs. miRNA + HPL | 0.7393 |
| 5G | Healthy vs. NT | 0.6075 |
| 5G | Healthy vs. miRNA | 0.5750 |
| 5G | Healthy vs. HPL | 0.9542 |
| 5G | Healthy vs. miRNA + HPL | 0.9623 |
| 5G | NT vs. HPL | 0.9448 |
| 5G | NT vs. miRNA + HPL | 0.9345 |
| 5G | miRNA  vs. HPL | 0.2136 |
| 5G | miRNA  vs. miRNA + HPL | 0.2279 |
| 5G | HPL vs. miRNA + HPL | >0.9999 |
| 6A | Healthy vs. NT | 0.6387 |
| 6A | Healthy vs. miRNA | 0.9573 |
| 6A | Healthy vs. HPL | 0.5351 |
| 6A | Healthy vs. miRNA + HPL | 0.9401 |
| 6A | NT vs. miRNA | 0.9684 |
| 6A | NT vs. HPL | 0.9998 |
| 6A | NT vs. miRNA + HPL | 0.2408 |
| 6A | miRNA  vs. HPL | 0.9299 |
| 6A | miRNA  vs. miRNA + HPL | 0.6365 |
| 6A | HPL vs. miRNA + HPL | 0.1811 |
| 6C | Healthy vs. NT | 0.7915 |
| 6C | Healthy vs. miRNA | 0.1346 |
| 6C | Healthy vs. HPL | 0.4814 |
| 6C | NT vs. miRNA | 0.6556 |
| 6C | NT vs. HPL | 0.9828 |
| 6C | NT vs. miRNA + HPL | 0.4841 |
| 6C | miRNA vs. HPL | 0.9169 |
| 6C | miRNA vs. miRNA + HPL | 0.9959 |
| 6C | HPL  vs. miRNA + HPL | 0.7779 |
| 6E | Healthy vs. miRNA | 0.5113 |
| 6E | Healthy vs. HPL | >0.9999 |
| 6E | Healthy vs. miRNA + HPL | 0.9003 |
| 6E | NT vs. miRNA | 0.1792 |
| 6E | miRNA vs. HPL | 0.5267 |
| 6E | miRNA vs. miRNA + HPL | 0.1336 |
| 6E | HPL  vs. miRNA + HPL | 0.8905 |
| 6G | Healthy vs. NT | 0.5824 |
| 6G | Healthy vs. miRNA | 0.9590 |
| 6G | Healthy vs. HPL | 0.9956 |
| 6G | Healthy vs. miRNA + HPL | 0.6715 |
| 6G | NT vs. miRNA | 0.9249 |
| 6G | NT vs. HPL | 0.7969 |
| 6G | miRNA  vs. HPL | 0.9979 |
| 6G | miRNA  vs. miRNA + HPL | 0.2943 |
| 6G | HPL vs. miRNA + HPL | 0.4505 |
| 6I | Healthy vs. NT | 0.1524 |
| 6I | Healthy vs. miRNA | 0.9953 |
| 6I | Healthy vs. HPL | 0.8757 |
| 6I | Healthy vs. miRNA + HPL | 0.6209 |
| 6I | NT vs. miRNA | 0.2862 |
| 6I | NT vs. HPL | 0.5934 |
| 6I | miRNA vs. HPL | 0.9785 |
| 6I | miRNA vs. miRNA + HPL | 0.3998 |
| 6I | HPL  vs. miRNA + HPL | 0.1652 |
| 6K | Healthy vs. NT | 0.9786 |
| 6K | Healthy vs. miRNA | 0.2727 |
| 6K | Healthy vs. HPL | 0.6501 |
| 6K | NT vs. miRNA | 0.1020 |
| 6K | NT vs. HPL | 0.3297 |
| 6K | miRNA vs. HPL | 0.9560 |
| 6K | miRNA vs. miRNA + HPL | 0.9510 |
| 6K | HPL  vs. miRNA + HPL | 0.6316 |
| 7A | Healthy vs. miRNA | 0.2707 |
| 7A | Healthy vs. HPL | 0.4611 |
| 7A | Healthy vs. miRNA + HPL | 0.4268 |
| 7A | miRNA vs. miRNA + HPL | 0.9976 |
| 7C | Healthy vs. miRNA | 0.5813 |
| 7C | Healthy vs. HPL | 0.4202 |
| 7C | Healthy vs. miRNA + HPL | 0.8473 |
| 7C | NT vs. HPL | 0.8306 |
| 7C | miRNA vs. miRNA + HPL | 0.9886 |
| 7E | Healthy vs. miRNA | 0.9479 |
| 7E | Healthy vs. HPL | 0.4166 |
| 7E | Healthy vs. miRNA + HPL | 0.9723 |
| 7E | NT vs. HPL | 0.4026 |
| 7E | miRNA vs. HPL | 0.1312 |
| 7E | miRNA vs. miRNA + HPL | >0.9999 |
| 7E | HPL  vs. miRNA + HPL | 0.1624 |
| 7G | Healthy vs. NT | 0.3787 |
| 7G | Healthy vs. miRNA | 0.8829 |
| 7G | Healthy vs. HPL | 0.9529 |
| 7G | Healthy vs. miRNA + HPL | 0.6825 |
| 7G | miRNA vs. HPL | 0.4949 |
| 7G | miRNA vs. miRNA + HPL | 0.9946 |
| 7G | HPL  vs. miRNA + HPL | 0.2903 |
| S2 A | Healthy vs. NT | 0.9388 |
| S2 A | Healthy vs. miRNA | 0.5868 |
| S2 A | Healthy vs. HPL | 0.9968 |
| S2 A | Healthy vs. miRNA + HPL | 0.9998 |
| S2 A | NT  vs. miRNA | 0.9406 |
| S2 A | NT  vs. HPL | 0.9927 |
| S2 A | NT  vs. miRNA + HPL | 0.9738 |
| S2 A | miRNA vs. HPL | 0.7720 |
| S2 A | miRNA vs. miRNA + HPL | 0.6787 |
| S2 A | HPL vs. miRNA + HPL | 0.9998 |
| S2 C | Healthy vs. NT | 0.9817 |
| S2 C | Healthy vs. miRNA | 0.9212 |
| S2 C | Healthy vs. HPL | >0.9999 |
| S2 C | Healthy vs. miRNA + HPL | 0.9071 |
| S2 C | NT  vs. miRNA | 0.9986 |
| S2 C | NT  vs. HPL | 0.9741 |
| S2 C | NT  vs. miRNA + HPL | 0.6283 |
| S2 C | miRNA  vs. HPL | 0.9029 |
| S2 C | miRNA  vs. miRNA + HPL | 0.4597 |
| S2 C | HPL vs. miRNA + HPL | 0.9250 |
| S2 E | Healthy vs. HPL | 0.9889 |
| S2 E | Healthy vs. miRDy | 0.6412 |
| S2 E | Healthy vs. miRDy + HPL | 0.5583 |
| S2 E | HPL  vs. miRDy | 0.8082 |
| S2 E | HPL  vs. miRDy + HPL | 0.7305 |
| S2 E | miRDy vs. miRDy + HPL | 0.9987 |
| S3 B | Healthy vs. NT | 0.9882 |
| S3 B | Healthy vs. miRNA | 0.8206 |
| S3 B | Healthy vs. HPL | 0.9998 |
| S3 B | Healthy vs. miRNA HPL | 0.9996 |
| S3 B | NT vs. miRNA | 0.5449 |
| S3 B | NT vs. HPL | 0.9976 |
| S3 B | NT vs. miRNA HPL | 0.9985 |
| S3 B | miRNA  vs. HPL | 0.7332 |
| S3 B | miRNA  vs. miRNA HPL | 0.7139 |
| S3 B | HPL vs. miRNA HPL | >0.9999 |
| S4 A | Healthy vs. miRNA | 0.9590 |
| S4 A | Healthy vs. HPL | >0.9999 |
| S4 A | Healthy vs. miRNA + HPL | 0.9592 |
| S4 A | NT  vs. miRNA | 0.1110 |
| S4 A | miRNA vs. HPL | 0.9756 |
| S4 A | miRNA vs. miRNA + HPL | 0.6619 |
| S4 A | HPL  vs. miRNA + HPL | 0.9368 |
| S4 C | Healthy vs. NT | >0.9999 |
| S4 C | Healthy vs. miRNA | 0.9965 |
| S4 C | Healthy vs. HPL | >0.9999 |
| S4 C | Healthy vs. miRNA + HPL | 0.9676 |
| S4 C | NT vs. miRNA | 0.9973 |
| S4 C | NT vs. HPL | >0.9999 |
| S4 C | NT vs. miRNA + HPL | 0.9716 |
| S4 C | miRNA  vs. HPL | 0.9967 |
| S4 C | miRNA  vs. miRNA + HPL | 0.9985 |
| S4 C | HPL vs. miRNA + HPL | 0.9688 |
